# Supplementary material for: Protective Effect of Alpinia oxyphylla Fruit against tert-Butyl Hydroperoxide-Induced Toxicity in HepG2 Cells via Nrf2 Activation and Free Radical Scavenging and Its Active Molecules
Source: Antioxidants (Basel). 2022 May 23;11(5):1032. doi: 10.3390/antiox11051032 (PMC9137508; doi:10.3390/antiox11051032)
Supplement: Supplementary file 1 [file antioxidants-11-01032-s001.zip › antioxidants-1725483-supplementary.pdf]

Supplementary Material

Protective Effect of *Alpinia oxyphylla* Fruits against *tert*-Butyl hydroperoxide Induced Toxicity in HepG2 Cells via Nrf2 Activation and Free Radical Scavenging

Chae Lee Park [1,2], Ji Hoon Kim [1], Je-Seung Jeon [1], Ju-hee Lee [1], Kaixuan Zhang [1], Shuo Guo [1], Do-hyun Lee [1], Eun Mei Gao [1], Rak Ho Son [1,2], Young-Mi Kim [1], Gyu Hwan Park [3,*], and Chul Young Kim [1,*]

1. Spectral Data of Isolated Compounds **1-12**

Figure S1. Isolation scheme of *n*-hexane extract of *A. oxyphylla*.
Figure S2. Isolation scheme of ethyl acetate extract.
Figure S3. ^{1}H-NMR spectrum of nootkatone (**1**) (400 MHz, CD_3OD).
Figure S4. ^{13}C-NMR spectrum of nootkatone (**1**) (100 MHz, CD_3OD).
Figure S5. ^{1}H-NMR spectrum of eudesma-3,11-dien-2-one (**2**) (400 MHz, $CDCl_3$).
Figure S6. ^{13}C-NMR spectrum of eudesma-3,11-dien-2-one (**2**) (100 MHz, $CDCl_3$).
Figure S7. ^{1}H-NMR spectrum of yakuchinone A (**3**) (400 MHz, $CDCl_3$).
Figure S8. ^{13}C-NMR spectrum of yakuchinone A (**3**) (100 MHz, $CDCl_3$).
Figure S9. ^{1}H-NMR spectrum of 5′-hydroxyl-yakuchinone A (**4**) (400 MHz, $CDCl_3$).
Figure S10. ^{1}H-NMR spectrum of 5′-hydroxyl-yakuchinone A (**4**) (300 MHz, CD_3OD).
Figure S11. ^{13}C-NMR spectrum of 5′-hydroxyl-yakuchinone A (**4**) (100 MHz, $CDCl_3$).
Figure S12. ^{1}H-^{1}H COSY spectrum of 5′-hydroxyl-yakuchinone A (**4**) (400 MHz, $CDCl_3$).
Figure S13. HSQC spectrum of 5′-hydroxyl-yakuchinone A (**4**) ($CDCl_3$).
Figure S14. HMBC spectrum of 5′-hydroxyl-yakuchinone A (**4**) ($CDCl_3$).
Figure S15. DEPE-135 spectrum of 5′-hydroxyl-yakuchinone A (**4**) ($CDCl_3$).
Figure S16. ESI-MS spectrum of 5′-hydroxyl-yakuchinone A (**4**).
Figure S17. ^{1}H-NMR spectrum of alpinenone (**5**) (500 MHz, $CDCl_3$).
Figure S18. ^{13}C-NMR spectrum of alpinenone (**5**) (125 MHz, $CDCl_3$).
Figure S19. ^{1}H-NMR spectrum of 6α–hydroxy-7-*epi*-α-cyperone (**6**) (400 MHz, CD_3OD).
Figure S20. ^{13}C-NMR spectrum of 6α–hydroxy-7-*epi*-α-cyperone (**6**) (100 MHz, CD_3OD).
Figure S21. ^{1}H-NMR spectrum of (4*S**,5*E*,10*R**)-7-oxo-tri-*nor*-eudesm-5-en-4β-ol (**7**) (400 MHz, $CDCl_3$).
Figure S22. ^{13}C-NMR spectrum of (4*S**,5*E*,10*R**)-7-oxo-tri-*nor*-eudesm-5-en-4β-ol (**7**) (100 MHz, $CDCl_3$).
Figure S23. ^{1}H-NMR spectrum of teuhetenone A (**8**) (400 MHz, $CDCl_3$).
Figure S24. ^{13}C-NMR spectrum of teuhetenone A (**8**) (100 MHz, $CDCl_3$).
Figure S25. ^{1}H-NMR spectrum of 7-*epi*-teucrenone B (**9**) (400 MHz, $CDCl_3$).
Figure S26. ^{13}C-NMR spectrum of 7-*epi*-teucrenone B (**9**) (100 MHz, $CDCl_3$).
Figure S27. ^{1}H-NMR spectrum of 11-hydroxyvalenc-1(10)-en-2-one (**10**) (400 MHz, $CDCl_3$).
Figure S28. ^{13}C-NMR spectrum of 11-hydroxyvalenc-1(10)-en-2-one (**10**) (100 MHz, $CDCl_3$).
Figure S29. ^{1}H-NMR spectrum of oxyphyllenodiol A (**11**) (400 MHz, $CDCl_3$).
Figure S30. ^{13}C-NMR spectrum of oxyphyllenodiol A (**11**) (100 MHz, $CDCl_3$).
Figure S31. ^{1}H-NMR spectrum of oxyphyllenodiol B (**11**) (400 MHz, $CDCl_3$).
Figure S32. ^{13}C-NMR spectrum of oxyphyllenodiol B (**11**) (100 MHz, $CDCl_3$).
Figure S33. HPLC chromatograms of crude extract and isolated compounds **1** – **12**.

Table S1. Partition coefficients of major peaks 1 and 2 of *A. oxyphylla* for CPC operation.
Table S2. Retention time and calibration curves of compounds **1** – **12**.

Spectral Data of Isolated Compounds 1 - 12

Nootkatone (**1**): $C_{15}H_{22}O$, ESI-MS: *m/z* 219.2 $[M+H]^+$, ^{1}H NMR (400 MHz, CD_3OD) δ 5.73 (1H, s, H-1), 4.71 (2H, s, H-12), 2.58 (1H, td, J = 14.8, 5.1 Hz), 2.43-2.28 (3H, m), 2.15 (1H, dd, J = 17.2, 3.8 Hz), 2.04-1.85 (1H, m), 1.39-1.27 (1H, m), 1.72 (3H, s, H-13), 1.13 (3H, s, H-15), 0.96 (3H, d, J = 6.8 Hz, H-14), 0.9-0.84 (1H, m, H-3,4,6,7,8,9)., ^{13}C-NMR (100 MHz, CD_3OD) δ 202.2 (C-2), 174.2 (C-10), 150.4 (C-11), 125.0 (C-1), 109.7 (C-12), 45.3 (C-9), 42.8 (C-3), 41.8 (C-7), 41.5 (C-4), 40.7 (C-5), 34.1 (C-6), 33.0 (C-8), 21.1 (C-13), 17.2 (C-15), 15.2 (C-14).

Eudesma-3,11-dien-2-one (**2**): $C_{15}H_{22}O$, ESI-MS: *m/z* 219.1 $[M+H]^+$, ^{1}H NMR (400 MHz, $CDCl_3$) δ 5.88 (1H, m, H-3), 4.99 (1H, dd, J = 2.9, 1.4 Hz, H-13a), 4.87 (1H, s, H-13b), 2.49 (2H, ddd, J = 10.2, 2.8, 1.4 Hz, H-5,7), 2.19 (2H, d, J = 3.0 Hz, H-1), 2.18-2.14 (1H, m, H-8a), 1.90 (3H, t, J = 1.4 Hz, H-15), 1.89-1.87 (1H, m, H-6a), 1.78 (3H, s, H-12), 1.76 (1H, s, H-6b), 1.58 (1H, s, H-9a), 1.56-1.53 (1H, m, H-8b), 1.31-1.28 (1H, m, H-9b), 0.93 (3H, s, H-14). ^{13}C NMR (100 MHz, $CDCl_3$) δ 199.5 (C-2), 163.7 (C-4), 146.0 (C-11), 127.2 (C-3), 111.7 (C-13), 54.9 (C-1), 42.9 (C-5), 39.1 (C-7), 38.2 (C-10), 35.9 (C-9), 25.2 (C-8), 22.9 (C-6), 22.8 (C-12), 22.1 (C-15), 16.7 (C-14).

Yakuchinone A (**3**): $C_{20}H_{24}O_3$, ESI-MS: *m/z* 313.4 $[M+H]^+$, ^{1}H NMR (400 MHz, $CDCl_3$) δ 7.27-7.23 (2H, m, H-3'', 5''), 7.16 (3H, dd, J = 12.0, 7.2 Hz, H-2'', 4'', 6''), 6.81 (1H, d, J = 7.9 Hz, H-5'), 6.68-6.63 (2H, m, H-2', 6'), 3.84 (3H, s, OCH_3), 2.81 (2H, t, J = 7.5 Hz, H-1), 2.67 (2H, t, J = 7.5 Hz, H-2), 2.59 (2H, t, J = 6.9 Hz, H-7), 2.39 (2H, t, J = 6.6 Hz, H-4), 1.58 (4H, dd, J = 6.7, 3.3 Hz, H-5,6). ^{13}C-NMR (100 MHz, $CDCl_3$) δ 210.4 (C-3), 146.5 (C-3'), 144.0 (C-4'), 142.3 (C-1''), 133.1 (C-1'), 128.5 (C-3'', 5''), 128.4 (C-2'', 6''), 125.9 (C-4''), 120.9 (C-6'), 114.4 (C-5'), 111.1 (C-2'), 56.0 (OCH3), 44.7 (C-2), 43.0 (C-4), 35.8 (C-7), 31.1 (C-6), 29.6 (C-1), 23.5 (C-5).

5′-Hydroxyl-yakuchinone A (**4**): $C_{20}H_{24}O_4$, ESI-MS: *m/z* 329.3 $[M+H]^+$, 351.4 $[M+Na]^+$, ^{1}H-NMR (400 MHz, CD_3OD) δ 7.23-7.18 (2H, m, H-3'',5''), 7.11 (3H, d, J = 7.0, H-2'',4'',6''), 6.78 (1H, d, J = 2.0 Hz, H-6'), 6.65 (1H, d, J = 2.0 Hz, H-2'), 3.86 (3H, s, OCH_3), 2.83-2.78 (2H, m, H-1), 2.77-2.71 (2H, m, H-2), 2.55 (2H, t, J = 7.1 Hz, H-7), 2.43 (2H, dd, J = 8.6, 5.1 Hz, H-4), 1.53 (4H, dt, J = 7.1, 3.6 Hz, H-5,6). ^{13}C-NMR (100 MHz, $CDCl_3$) δ 210.4 (C-3), 147.3 (C-3'), 142.3 (C-4'), 141.0 (C-1''), 133.2 (C-1'), 128.5 (C-3'', 5''), 128.4 (C-2'', 6''), 125.9 (C-4''), 124.5 (C-6'), 122.8 (C-5'), 110.8 (C-2'), 56.2 (OCH3), 44.7 (C-2), 43.0 (C-4), 35.9 (C-7), 31.1 (C-6), 29.7 (C-1), 23.5 (C-5).

Alpinenone (**5**): $C_{15}H_{22}O_3$, ^{1}H-NMR (500 MHz, $CDCl_3$) δ 5.90 (1H, s, H-8), 2.91 (1H, dd, J = 13.5, 6.8 Hz, H-11), 2.88-2.83 (1H, m, H-1), 2.80-2.74 (1H, m, H-5), 2.03 (1H, dd, J = 13.8, 6.8 Hz, H-4), 1.44 (3H, s, H-15), 1.40-1.34 (2H, m), 1.40-1.34 (2H, m, H-2a), 1.22 (3H, d, J = 6.6 Hz, H-13), 1.19 (1H, s, H-2b), 1.15 (3H, d, J = 6.8 Hz, H-12), 1.08 (3H, d, J = 7.2 Hz, H-14). ^{13}C-NMR (125 MHz, $CDCl_3$) δ 199.3 (C-9), 176.5 (C-7), 123.6(C-8), 105.5 (C-6), 88.2 (C-10), 63.1 (C-5), 52.5(C-1), 36.4(C-4), 33.0 (C-2), 30.1 (C-11), 25.7 (C-13), 24.6 (C-3), 20.8 (C-15), 20.7 (C-12), 14.5 (C-14).

6α–Hydroxy-7-*epi*-α-cyperone (**6**): $C_{15}H_{22}O_2$, ^{1}H-NMR (400 MHz, CD_3OD) δ 4.84 (1H, s, H-6), 4.80 (1H, s, H-12α), 4.39 (1H, s, H-12β), 2.65 (1H, m, H-2α), 2.48 (1H, s, H-7), 2.34 (1H, dd, J = 17.8, 3.3 Hz, H-2β), 2.22 (1H, m, 8α), 1.83 (3H, s, H-15), 1.77 (1H, m, H-1β), 1.73 (3H, s, H-13), 1.61 (1H, dd, J = 12.9, 4.5, H-1α), 1.53-1.44 (3H, dd, J = 25.5, 13.6 Hz, H-8b. H-9a, H9b), 1.38 (3H, s, H-14). ^{13}C-NMR (100 MHz, CD_3OD) δ 202.1 (C-3), 162.8 (C-5), 147.0 (C-11), 132.6 (C-4), 111.8 (C-12), 69.8 (C-6), 49.2 (C-7), 40.0 (C-1), 36.3 (C-9), 36.3 (C-10), 35.0 (C-2), 25.7 (C-14), 23.4 (C-13), 19.5 (c-8), 10.6 (C-15).

(4*S**,5*E*,10*R**)-7-Oxo-tri-nor-eudesm-5-en-4β-ol (**7**): $C_{12}H_{18}O_2$, ^{1}H-NMR (400 MHz, $CDCl_3$) δ 6.03 (1H, s, H-6), 2.65-2.54 (1H, m, H-8a), 2.39 (1H, d, J = 17.6 Hz, H-8b), 2.16-2.02 (1H, m, H-2a), 1.93 (1H, d, J = 12.4 Hz, H-3a), 1.85 (1H, dd, J = 14.6, 4.4 Hz, H-9a), 1.70 (1H, d, J = 3.8 Hz, H-9b), 1.67 (1H, d, J = 2.5 Hz, H-1a), 1.54 (1H, d, J = 4.2, H-2b), 1.51 (1H, s, H-3b), 1.45 (3H, s, H-11), 1.42 (3H, s, H-12), 1.34 (1H,

td, J = 13.4, 3.1 Hz, H-1b). ^{13}C-NMR (100 MHz, $CDCl_3$) δ 201.4 (C-7), 170.0 (C-5), 123.4 (C-6), 71.6 (C-4), 41.4 (C-1), 40.4 (C-3), 40.3 (C-9), 36.0 (C-10), 34.2 (C-8), 29.2 (C-12), 24.6 (C-11), 17.4 (C-2).

Teuhetenone A (**8**): $C_{12}H_{18}O_2$, ^{1}H-NMR (400 MHz, $CDCl_3$) δ 6.35 (1H, s, H-6), 2.62-2.52 (1H, m, H-9a), 2.38 (1H, d, J = 17.7 Hz, H-9b), 1.98 (1H, d, J = 12.9, H-8a), 1.89 (1H, dd, J = 14.3 Hz, H-3a), 1.78 (1H, d, J = 5.1 Hz, H-1a), 1.75-1.70 (2, m, H-2), 1.66 (1H, d, J = 15.3 Hz, H-3b), 1.56 (1H, td, J = 12.5, 5.2 Hz, H-8b), 1.43 (3H, s, H-12), 1.40-1.33 (1H, m, H-1b), 1.31 (3H, s, H-11). ^{13}C-NMR (100 MHz, $CDCl_3$) δ 200.6 (C-7), 175.2 (C-5), 122.7 (C-6), 72.5 (C-4), 42.4 (C-8), 41.0 (C-3), 40.8 (C-1), 36.3 (C-10), 34.1 (C-9), 29.8 (C-12) 24.7 (C-11), 19.6 (C-2).

7-*epi*-Teucrenone B (**9**): $C_{15}H_{22}O_2$, ^{1}H-NMR (400 MHz, $CDCl_3$) δ 5.89 (1H, s, H-3), 5.10 (2H, s, H-12), 2.36 (1H, d, J = 13.1 Hz, H-6a), 2.31 (1H, s, H-5), 2.27 (1H, s, H-1a), 2.14 (1H, s, H-1b), 2.11 (1H, dd, J = 12.7, 4.0 Hz, H-8a), 1.93 (3H, s, H-15), 1.85 (3H, s, H-13), 1.82-1.73 (1H, m, H-8b), 1.57 (1H, d, J = 13.1 Hz, H-6b), 1.48 (2H, dd, J = 7.9, 3.7 Hz, H-9), 0.94 (3H, s, H-14). ^{13}C-NMR (100 MHz, $CDCl_3$) δ 199.1 (C-2), 162.3 (C-4), 146.2 (C-11), 127.1 (C-3), 114.3 (C-12), 74.9 (C-7), 54.3 (C-1), 45.0 (C-5), 37.8 (C-9), 37.6 (C-10), 33.2 (C-6), 31.7 (C-8), 22.1 (C-15), 18.8 (C-13), 17.1 (C-14).

11-Hydroxyvalenc-1(10)-en-2-one (**10**): $C_{15}H_{24}O_2$, ^{1}H-NMR (500 MHz, $CDCl_3$) δ 5.75 (1H, s, H-1), 2.46 (1H, dd, J = 5.2, 1.7 Hz, H-9a), 2.38 (1H, dd, J = 4.1, 2.7 Hz, H-9b), 2.23 (2H, d, J = 4.5 Hz, H-3), 2.03-2.01 (1H, m, H-6a), 2.01-1.98 (1H, m, H-8), 1.96-1.95 (1H, m, H-4), 1.71 (1H, dd, J = 6.6, 3.6 Hz, H-7), 1.19 (3H, s, H-12), 1.17 (3H, s, H-13), 1.07 (3H, s, H-15), 0.97 (1H, s, H-6b), 0.96 (3H, d, J=6.8 Hz, H-14). ^{13}C-NMR (125 MHz, $CDCl_3$) δ 200.3 (C-2), 171.5(C-10), 124.5 (C-1), 72.6 (C-11), 43.9 (C-7), 42.1 (C-3), 40.6 (C-4), 39.7 (C-6), 39.3 (C-5), 33.1 (C-9), 27.8 (C-8), 27.4 (C-12), 26.9 (C-13), 17.0 (C-15), 15.1 (C-14).

Oxyphyllenodiol A (**11**): $C_{14}H_{22}O_3$, ^{1}H-NMR (400 MHz, $CDCl_3$) δ 4.16 (1H, br s, H-4), 2.66-2.60 (1H, m, H-6), 2.56-2.50 (1H, m, H-8a), 2.50-2.45 (1H, m, H-1a), 2.33 (1H, t, J = 6.1 Hz, H-8b), 2.29 (1H, t, J =6.2, H-1b), 2.22-2.17 (1H, m, H-11), 1.99-1.93 (1H, m, H-7), 1.78 (1H, ddd, J = 13.4, 8.4, 6.5 Hz, H-2a), 1.66 (1H, ddd, J = 13.3, 6.3, 5.3 Hz, H-2b), 1.22 (3H, s, H-14), 1.06 (3H, d, J = 6.8 Hz, H-13), 0.91 (3H, d, J = 6.9 Hz, H-12). ^{13}C-NMR (100 MHz, $CDCl_3$) δ 199.9 (C-9), 157.5 (C-5), 132.7 (C-10), 75.3 (C-4), 72.3 (C-3), 40.3 (C-6), 35.1 (C-8), 32.2 (C-2), 30.0 (C-11), 22.5 (C-7), 22.1 (C-14), 21.7 (C-13), 21.6 (C-1), 19.3 (C-12).

Oxyphyllenodiol B (**12**) : $C_{14}H_{22}O_3$, ^{1}H-NMR (400 MHz, $CDCl_3$) δ 3.96 (1H, d, J = 5.8 Hz, H-4), 2.64-2.58 (1H, m, H-6), 2.51 (1H, ddd, J = 17.2, 8.8, 5.1 Hz, H-8a), 2.34 (2H, dd, J = 8.3, 5.7 Hz, H-1), 2.3 (1H, s, H-8b), 2.27-2.20 (1H, m, H-11), 2.11 (1H, s, J = 5.2 Hz, H-7a), 1.99 (1H, ddd, J = 13.9, 5.3, 3.4 Hz, H-7b), 1.90-1.80 (1H, m, H-2a), 1.59 (1H, dt, J = 13.2, 9.2 Hz, H-2b), 1.27 (3H, s, H-14), 1.04 (3H, d, J = 6.8 Hz, H-13), 0.85 (3H, d, J = 6.9 Hz, H-12). ^{13}C-NMR (100 MHz, $CDCl_3$) δ 199.9 (C-9), 156.5 (C-5), 133.5 (C-10), 72.5 (C-4), 70.3 (C-3), 41.3 (C-6), 35.6 (C-8), 31.3 (C-2), 29.2 (C-11), 25.2 (C-14), 22.0 (C-7), 21.5 (C-13), 20.8 (C-1), 18.6 (C-12).

Table S1. Partition coefficients of major peaks 1 and 2 of *A. oxyphylla* for CPC operation.

Solvent systems	K values of the peaks 1 and 2	
n-hexane/EtOAc/methanol/water, v/v/v/v)	Peak 1	Peak 2
9:1:9:1	0.10	0.59
8:2:8:2	0.32	0.15
**7:3:7:3**	**0.57**	**2.22**
6:4:6:4	1.33	4.32
6:4:6:4	1.69	3.34
7:3:5:5	2.89	4.79

EtOAc: ethyl acetate

After considering *K* values of peaks **1** and **2**, two-phase solvent system with hexane/EtOAc/methanol/water (7:3:7:3, v/v/v/v) was chosen for CPC operation. Peaks **1** and **2** were marked in Figure S1B.

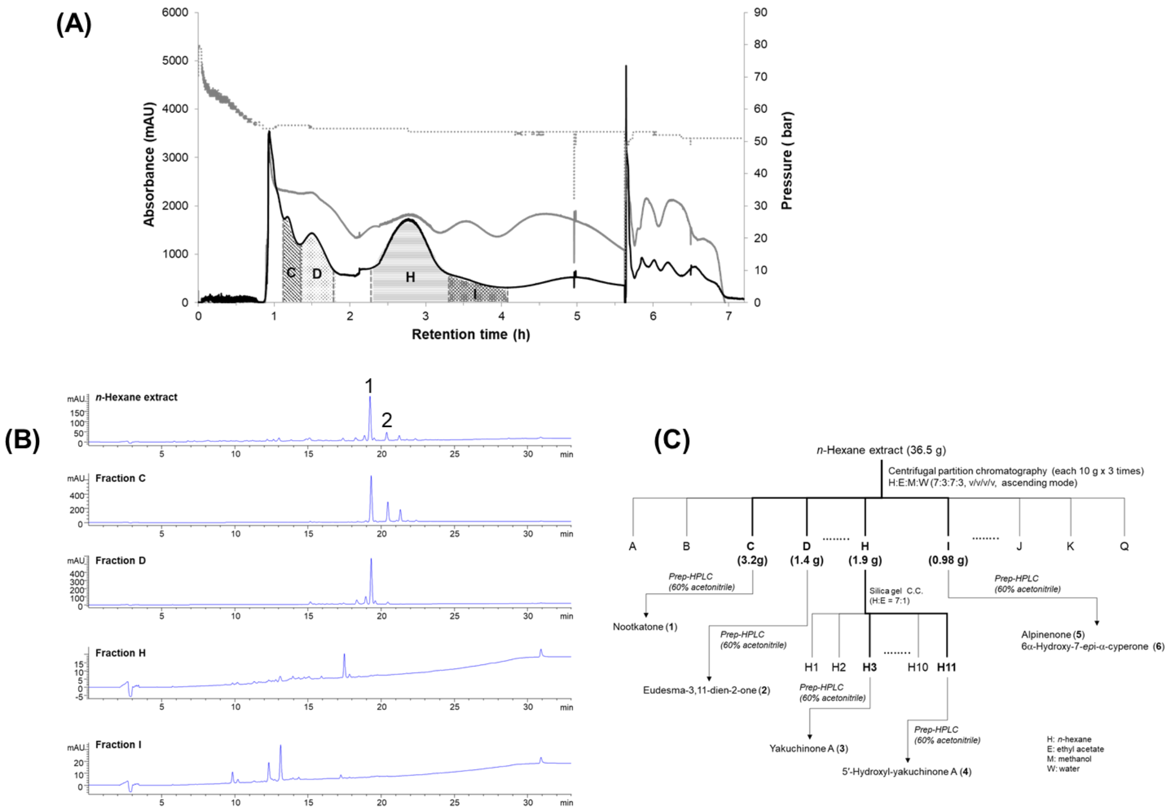

Figure S1. Isolation scheme of *n*-hexane extract of *A. oxyphylla*. (A) CPC chromatogram of *n*-hexane extract of *A. oxyphylla*, (B) HPLC chromatograms fractions C, D, H, and I obtained from CPC and (C) Isolation scheme of *n*-hexane extract. CPC and HPLC operation conditions were described in Materials and Methods Section.

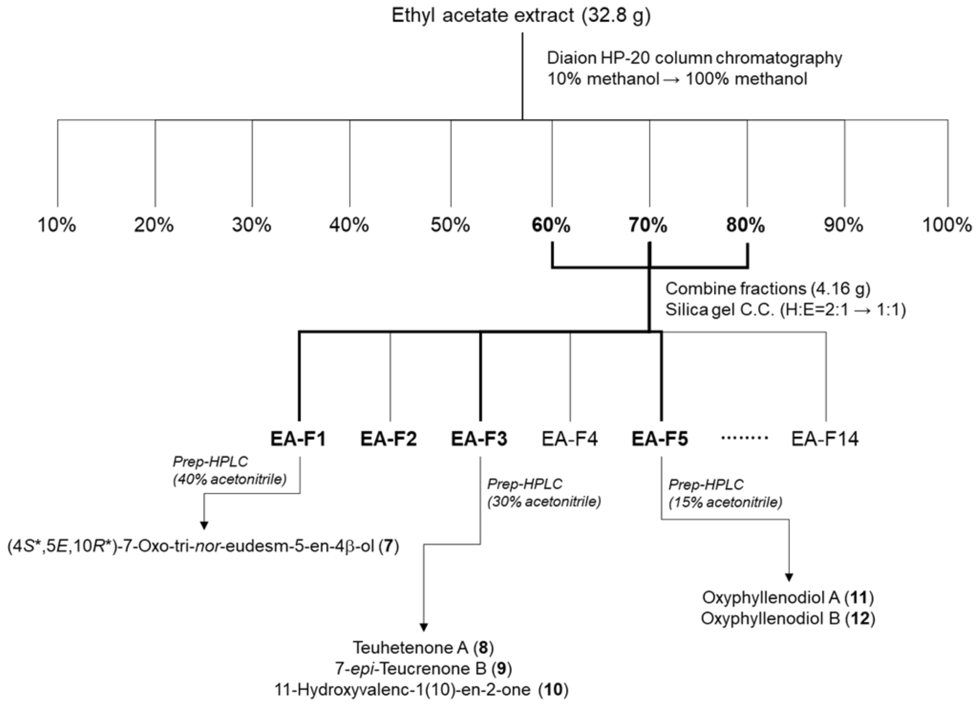

Figure S2. Isolation scheme of ethyl acetate extract.

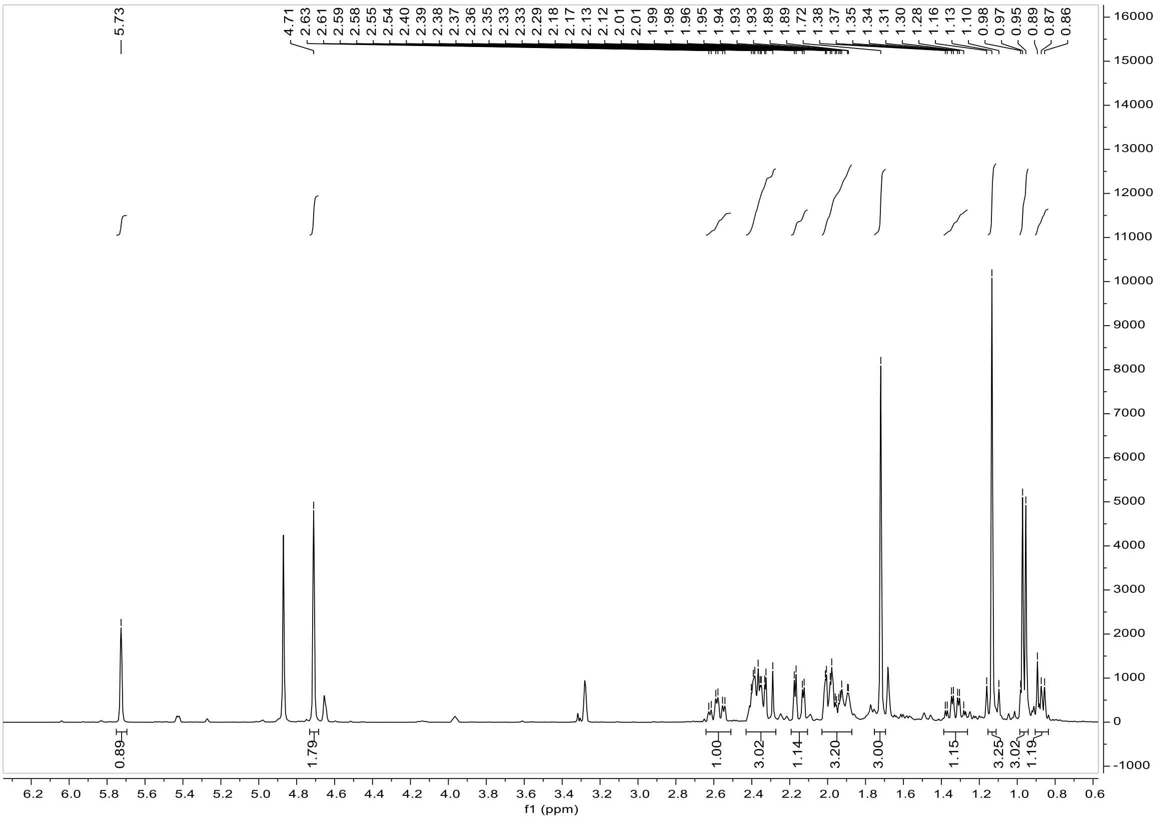

Figure S3. ^{1}H-NMR spectrum of nootkatone (**1**) (400 MHz, CD_3OD).

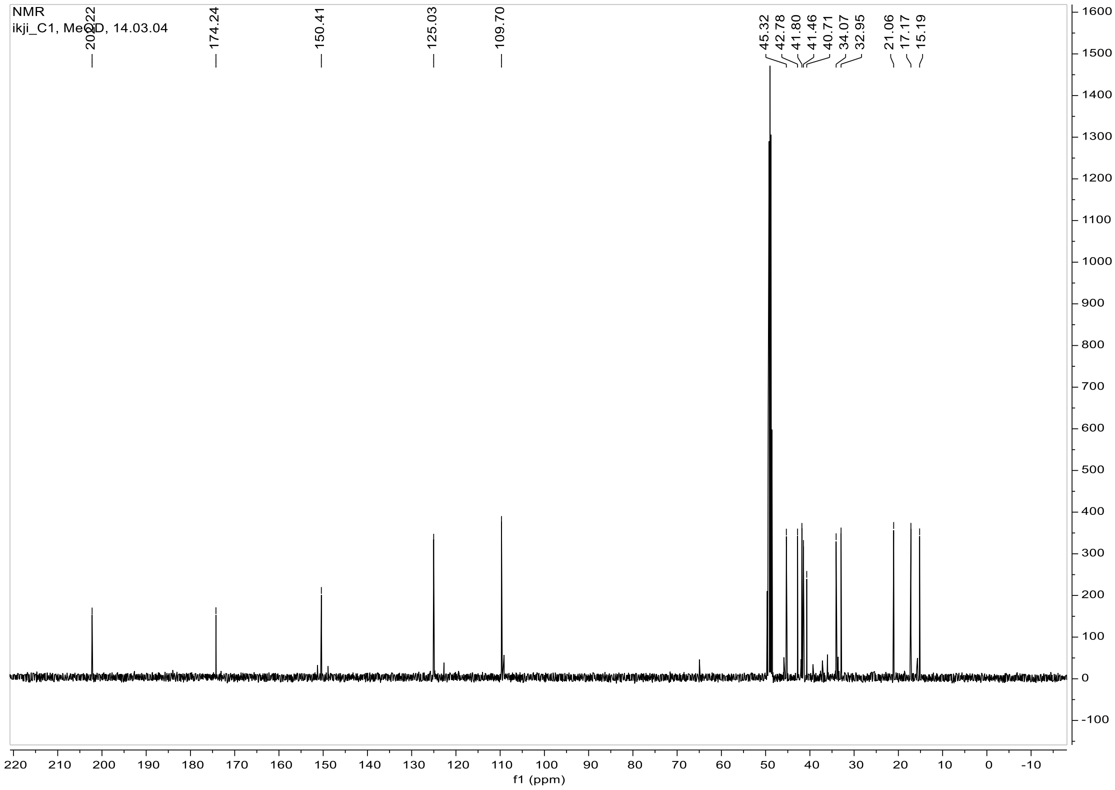

Figure S4. ^{13}C-NMR spectrum of nootkatone (**1**) (100 MHz, CD_3OD).

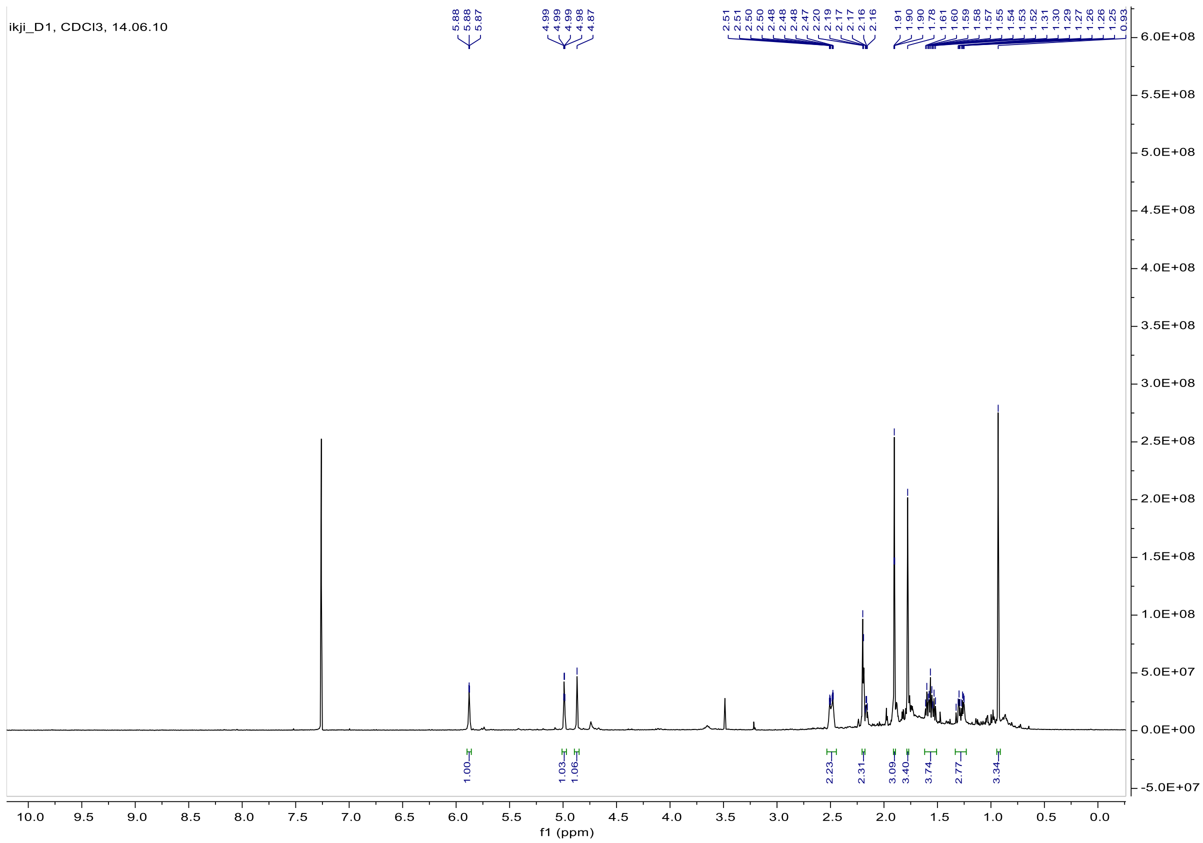

Figure S5. ^{1}H-NMR spectrum of eudesma-3,11-dien-2-one (**2**) (400 MHz, $CDCl_3$).

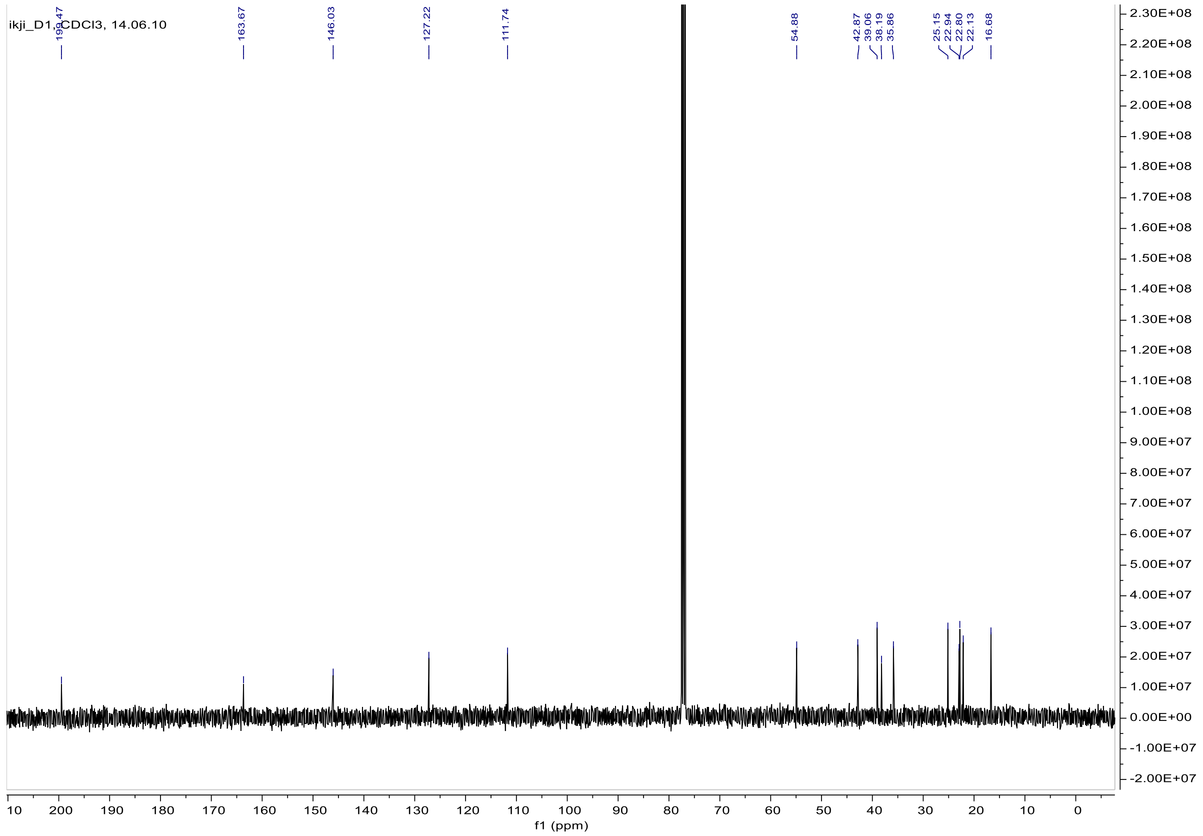

Figure S6. ^{13}C-NMR spectrum of eudesma-3,11-dien-2-one (**2**) (100 MHz, $CDCl_3$).

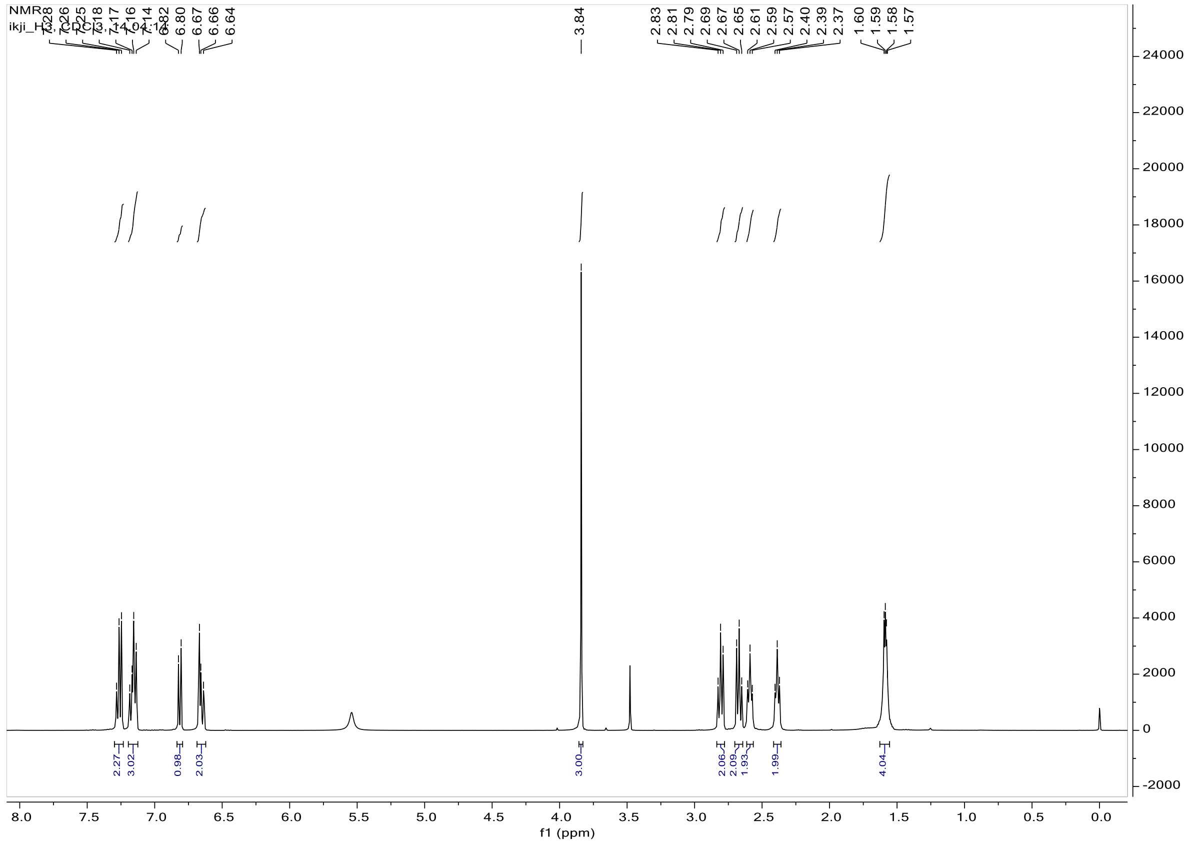

Figure S7. ^{1}H-NMR spectrum of yakuchinone A (**3**) (400 MHz, $CDCl_3$).

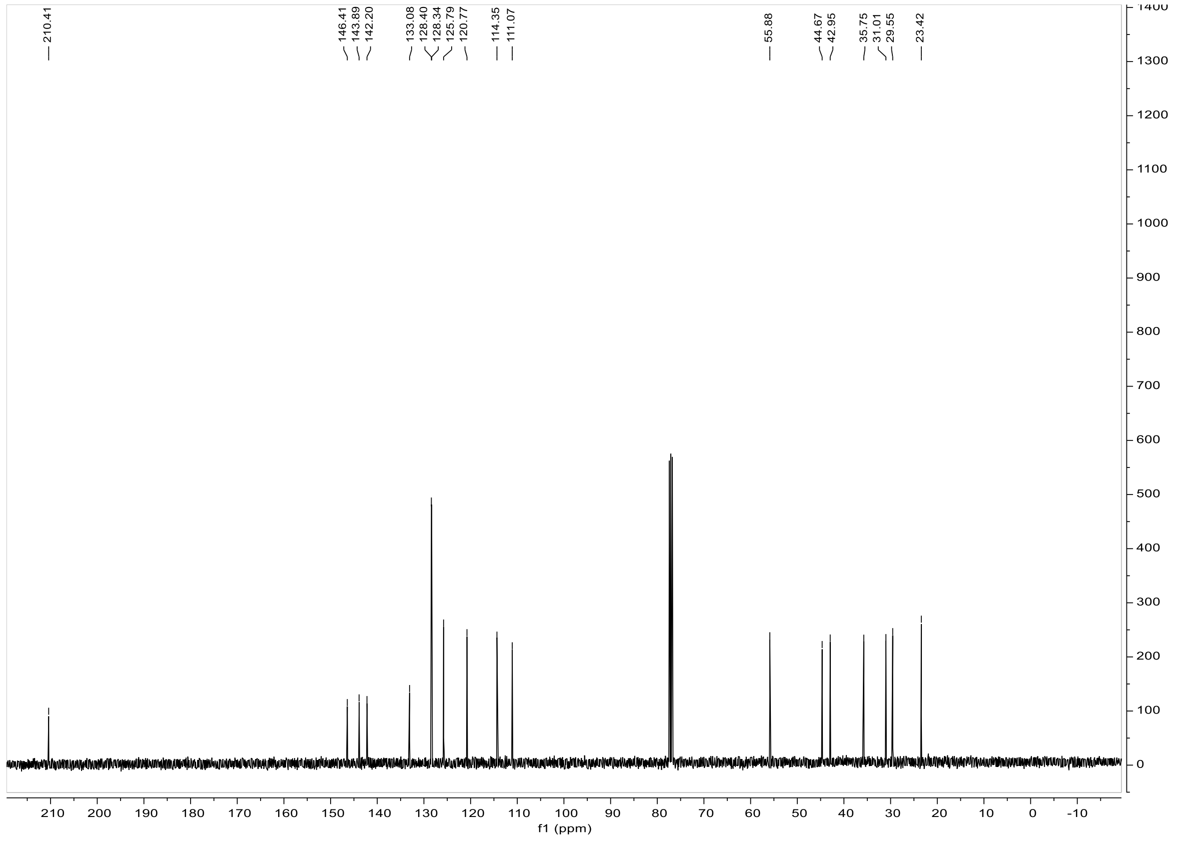

Figure S8. ^{13}C-NMR spectrum of yakuchinone A (**3**) (100 MHz, $CDCl_3$).

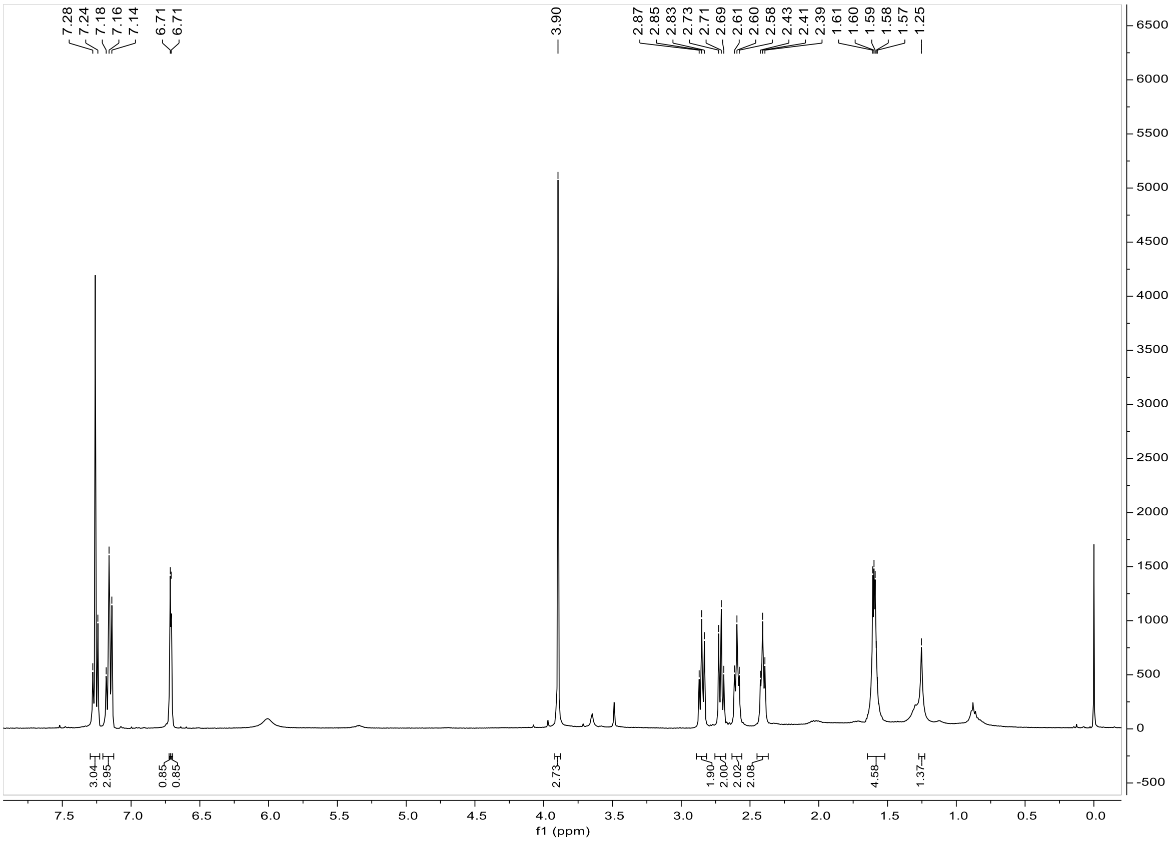

Figure S9. ^{1}H-NMR spectrum of 5′-hydroxyl-yakuchinone A (**4**) (400 MHz, $CDCl_3$).

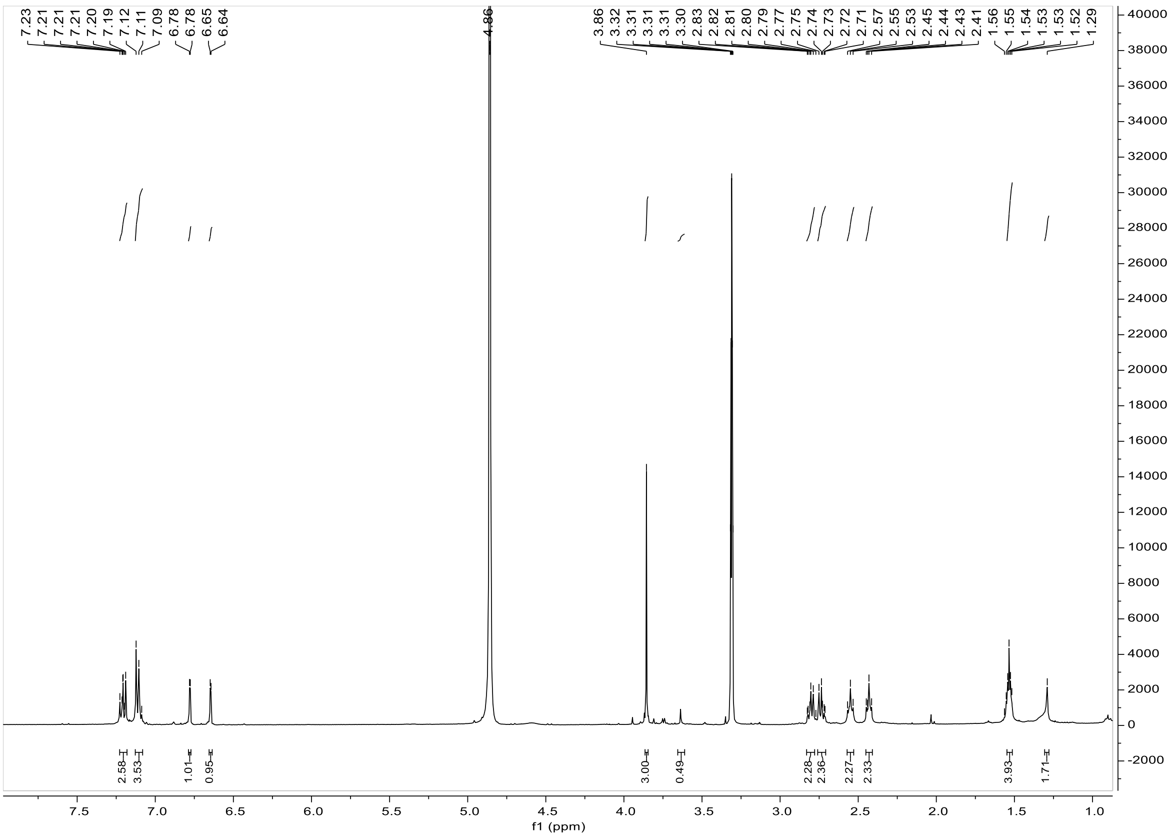

Figure S10. ^{1}H-NMR spectrum of 5′-hydroxyl-yakuchinone A (**4**) (300 MHz, CD_3OD).

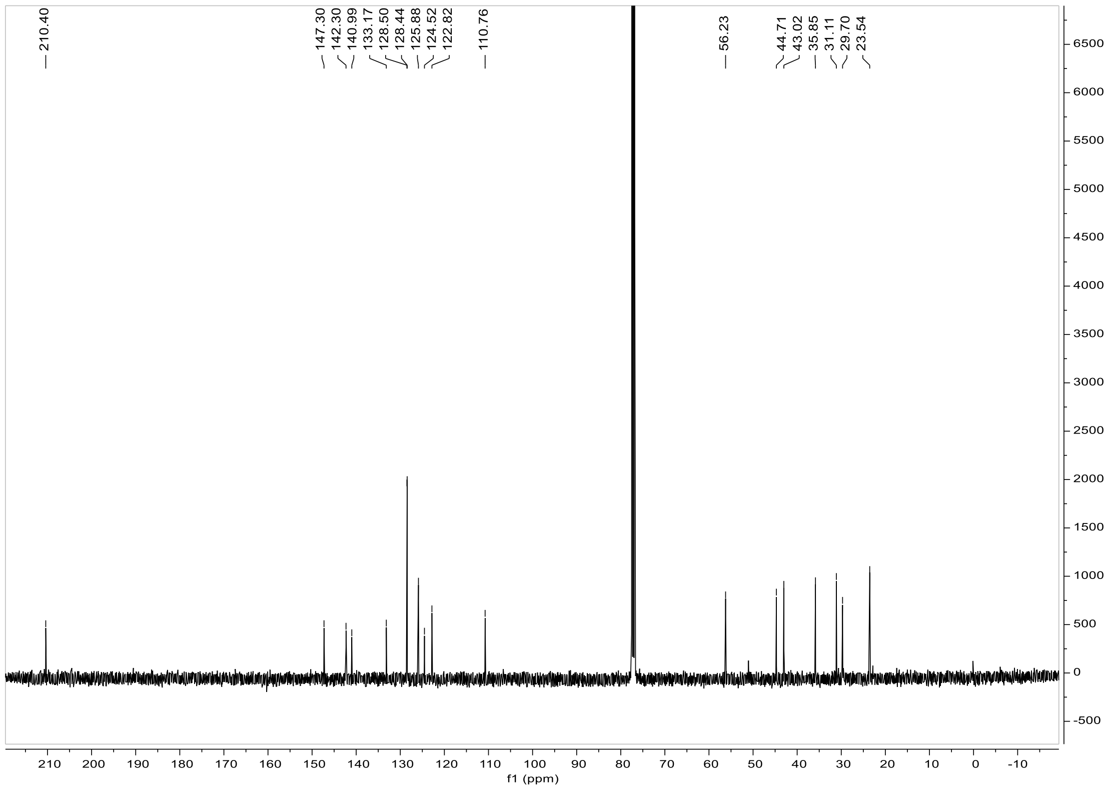

Figure S11. ^{13}C-NMR spectrum of 5′-hydroxyl-yakuchinone A (**4**) (100 MHz, $CDCl_3$).

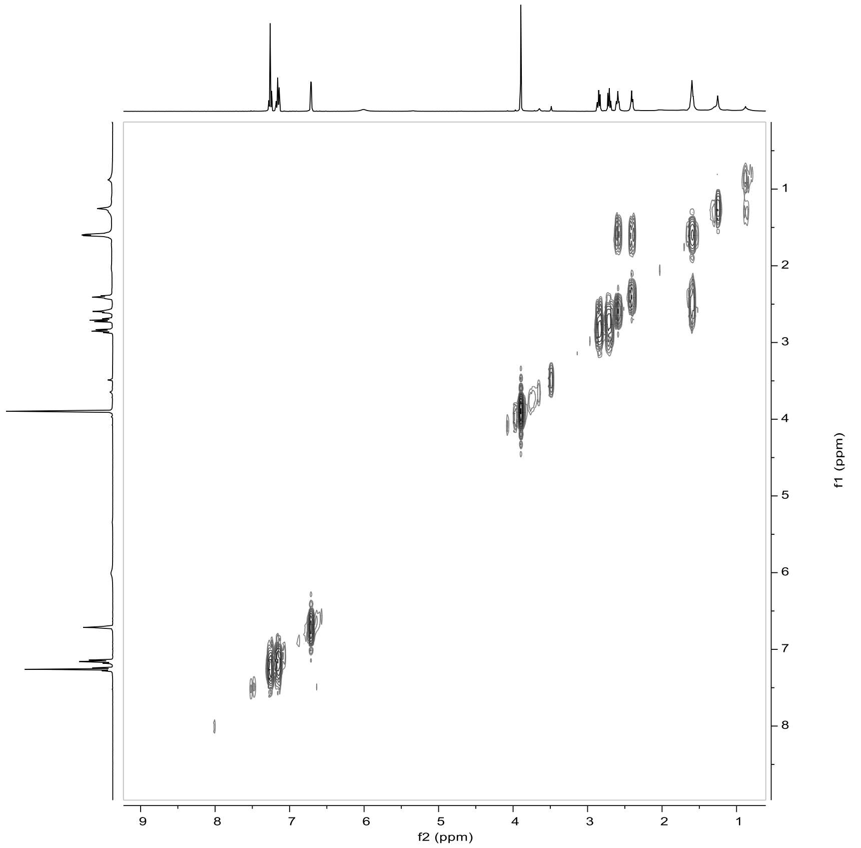

Figure S12. ^{1}H-^{1}H COSY spectrum of 5′-hydroxyl-yakuchinone A (**4**) (400 MHz, $CDCl_3$).

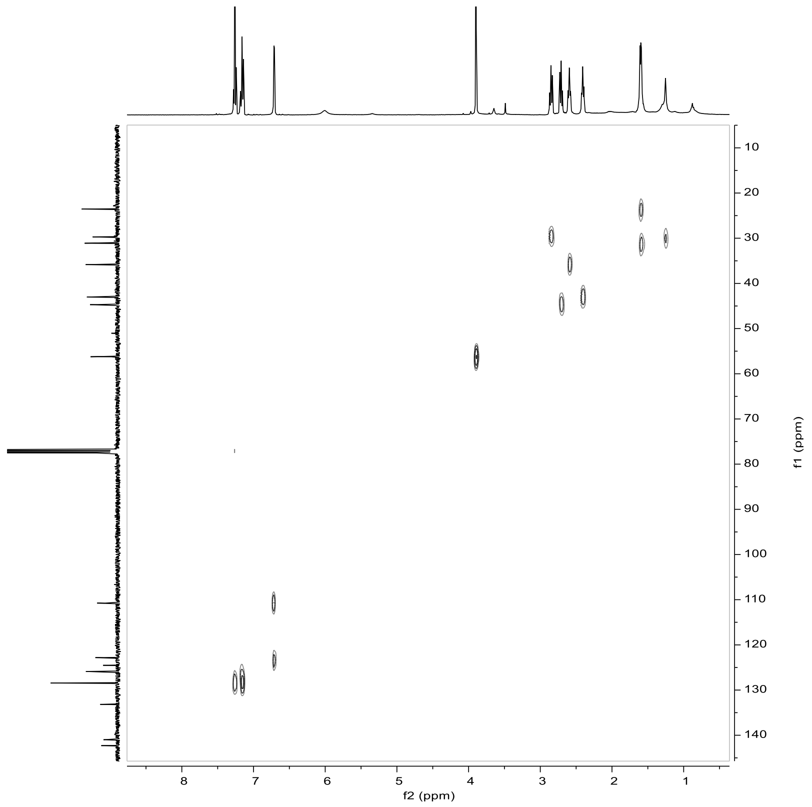

Figure S13. HSQC spectrum of 5′-hydroxyl-yakuchinone A (**4**) ($CDCl_3$).

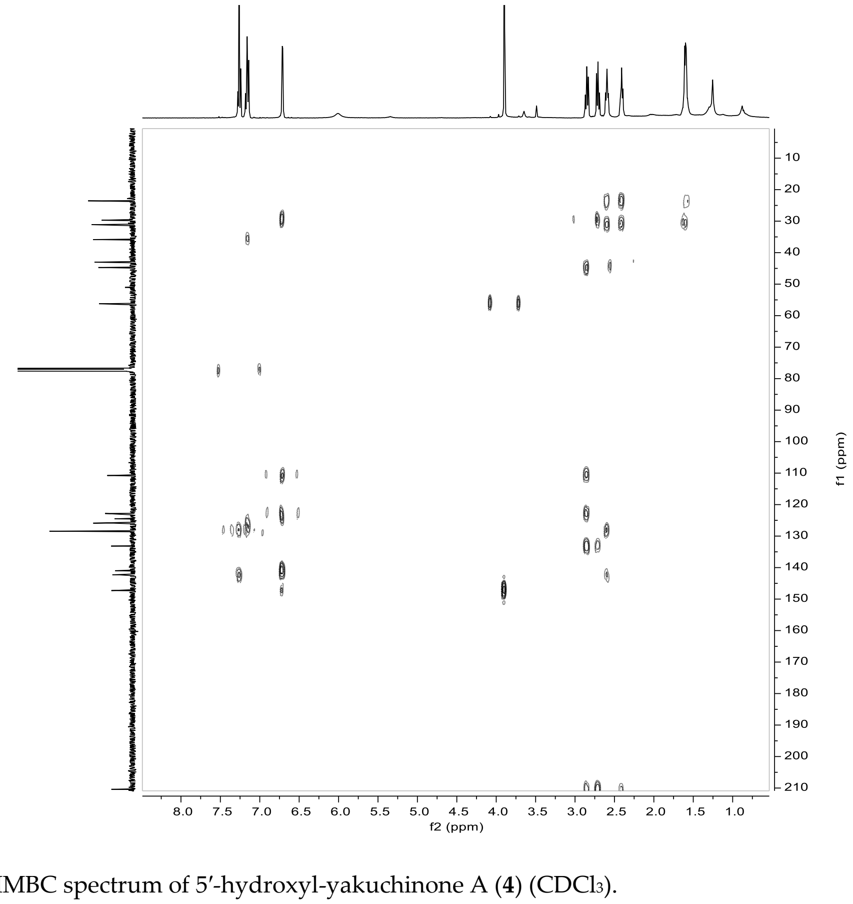

Figure S14. HMBC spectrum of 5′-hydroxyl-yakuchinone A (**4**) ($CDCl_3$).

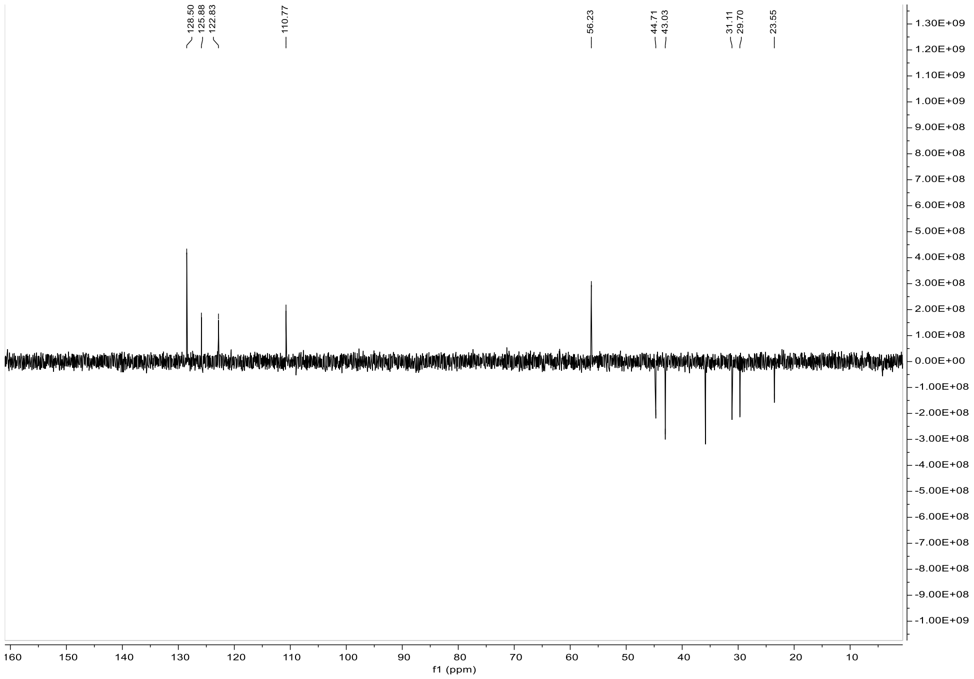

Figure S15. DEPE-135 spectrum of 5′-hydroxyl-yakuchinone A (**4**) ($CDCl_3$).

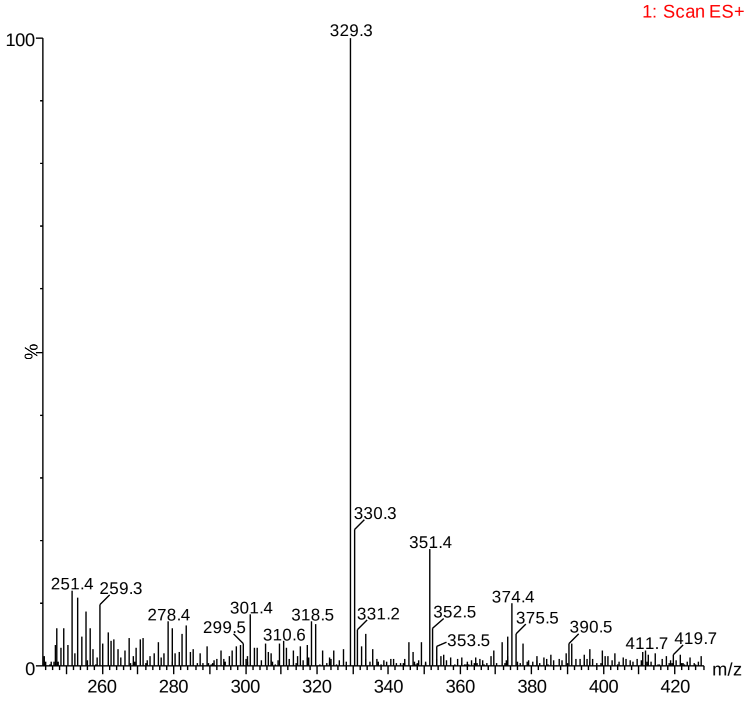

Figure S16. ESI-MS spectrum of 5′-hydroxyl-yakuchinone A (**4**).

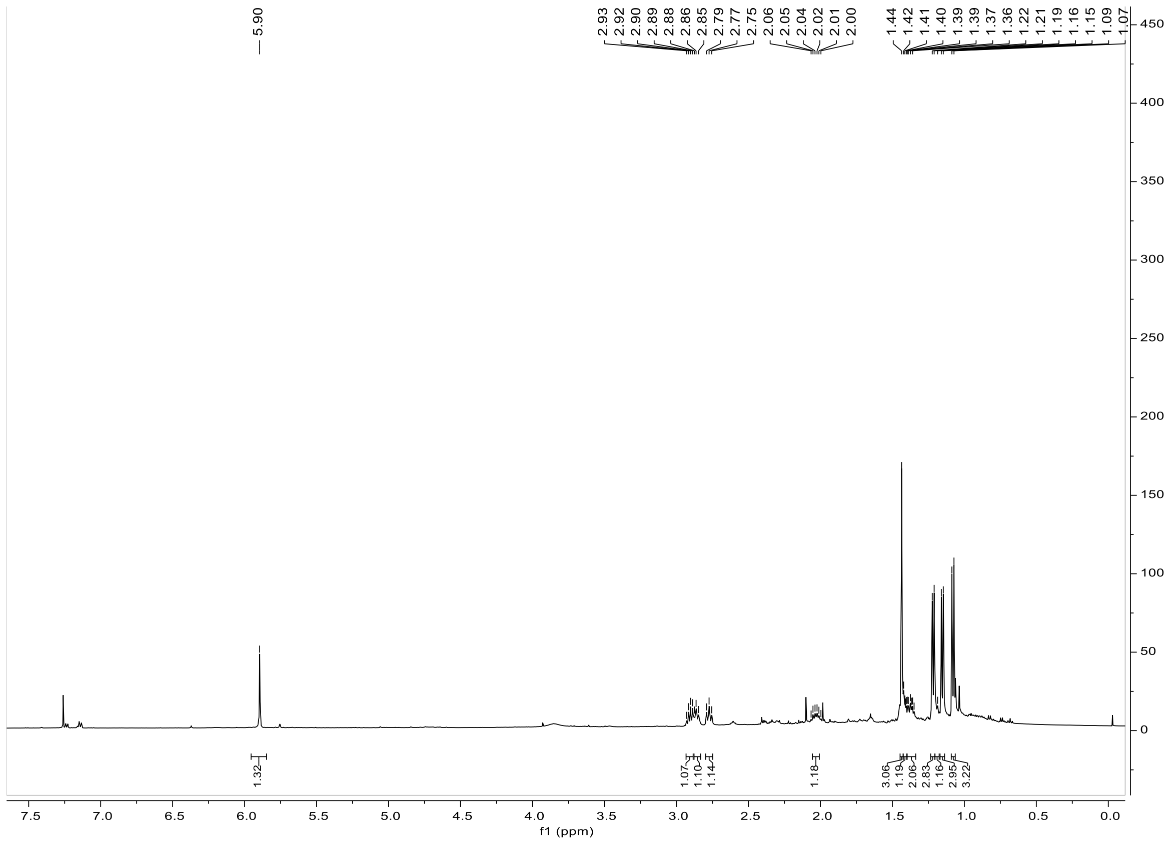

Figure S17. ^{1}H-NMR spectrum of alpinenone (**5**) (500 MHz, $CDCl_3$).

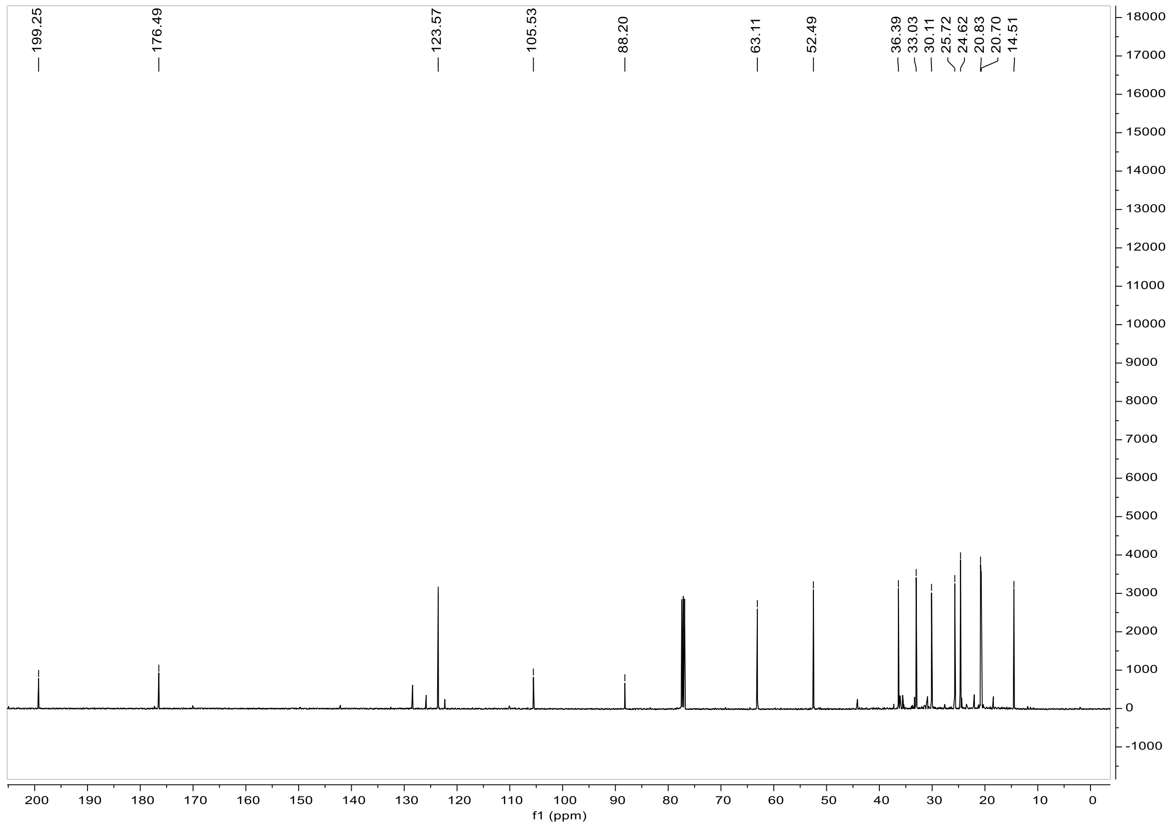

Figure S18. ^{13}C-NMR spectrum of alpinenone (**5**) (125 MHz, $CDCl_3$).

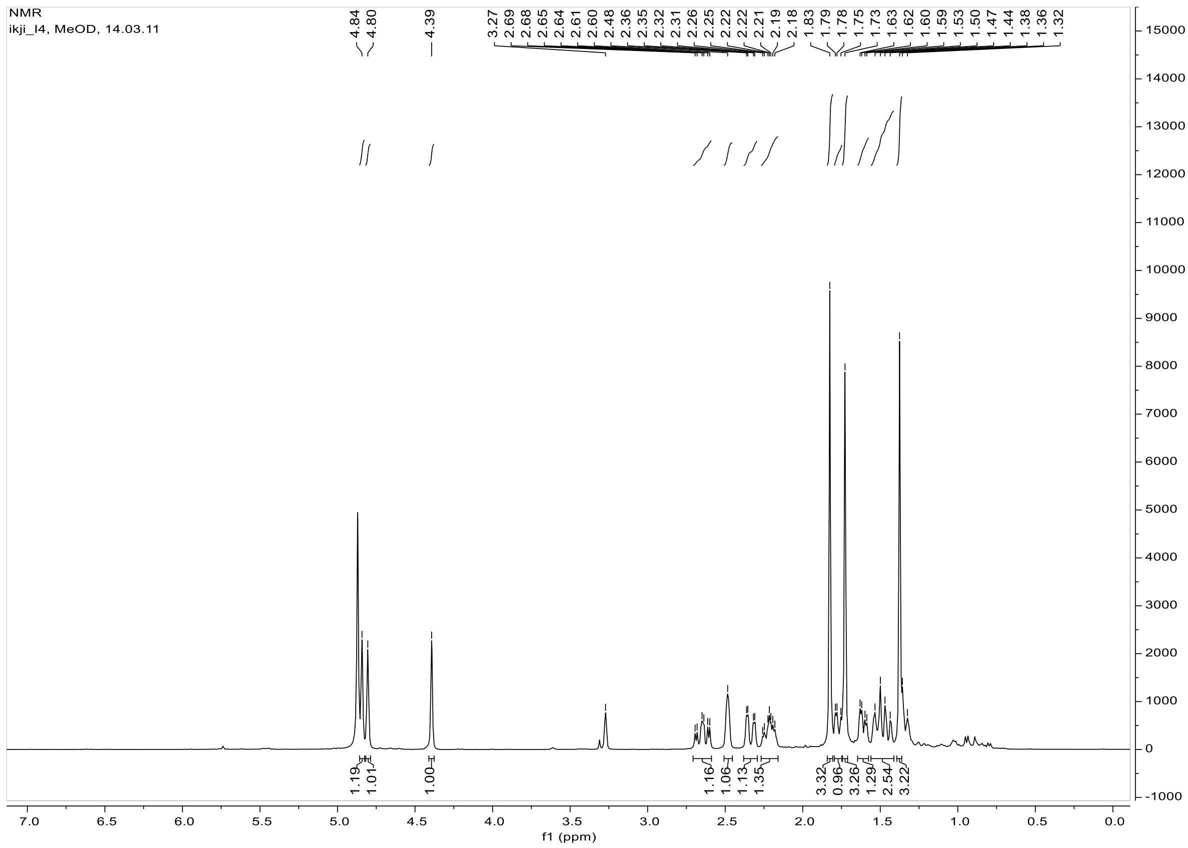

Figure S19. ^{1}H-NMR spectrum of 6α–hydroxy-7-*epi*-α-cyperone (**6**) (400 MHz, CD_3OD).

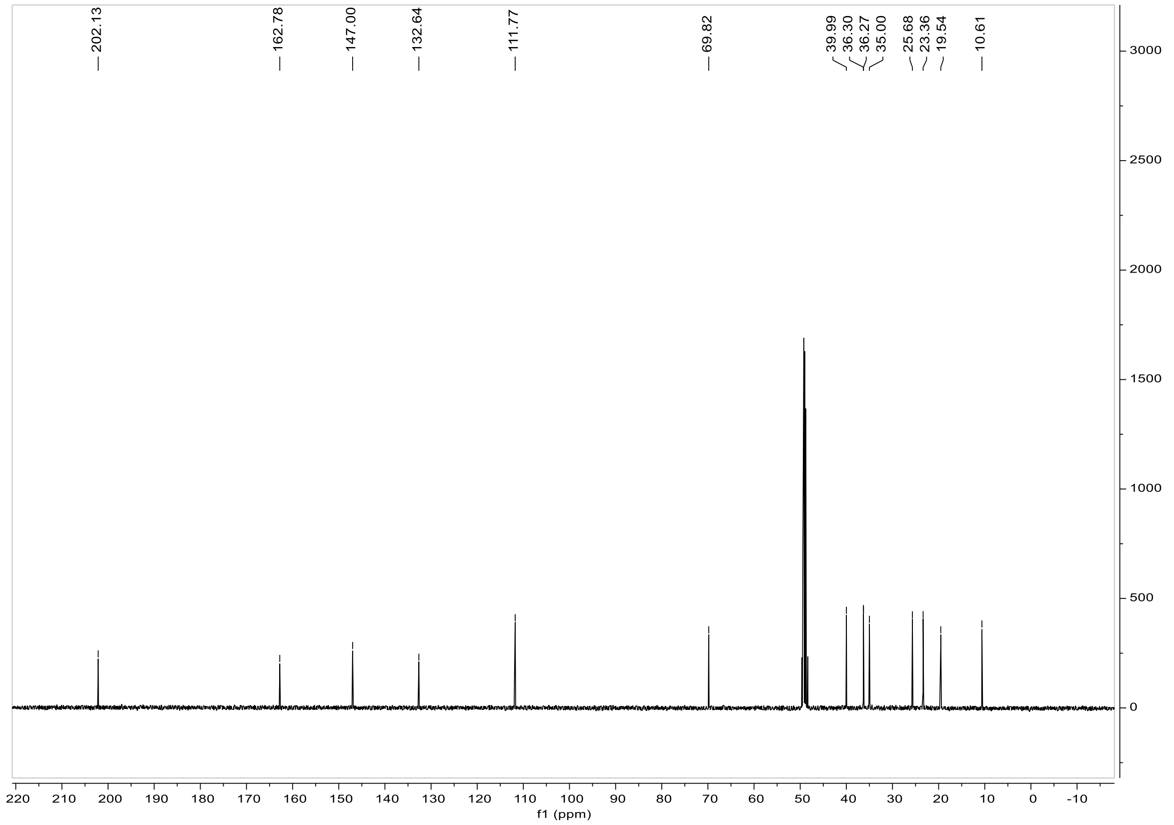

Figure S20. ^{13}C-NMR spectrum of 6α–hydroxy-7-*epi*-α-cyperone (**6**) (100 MHz, CD_3OD).

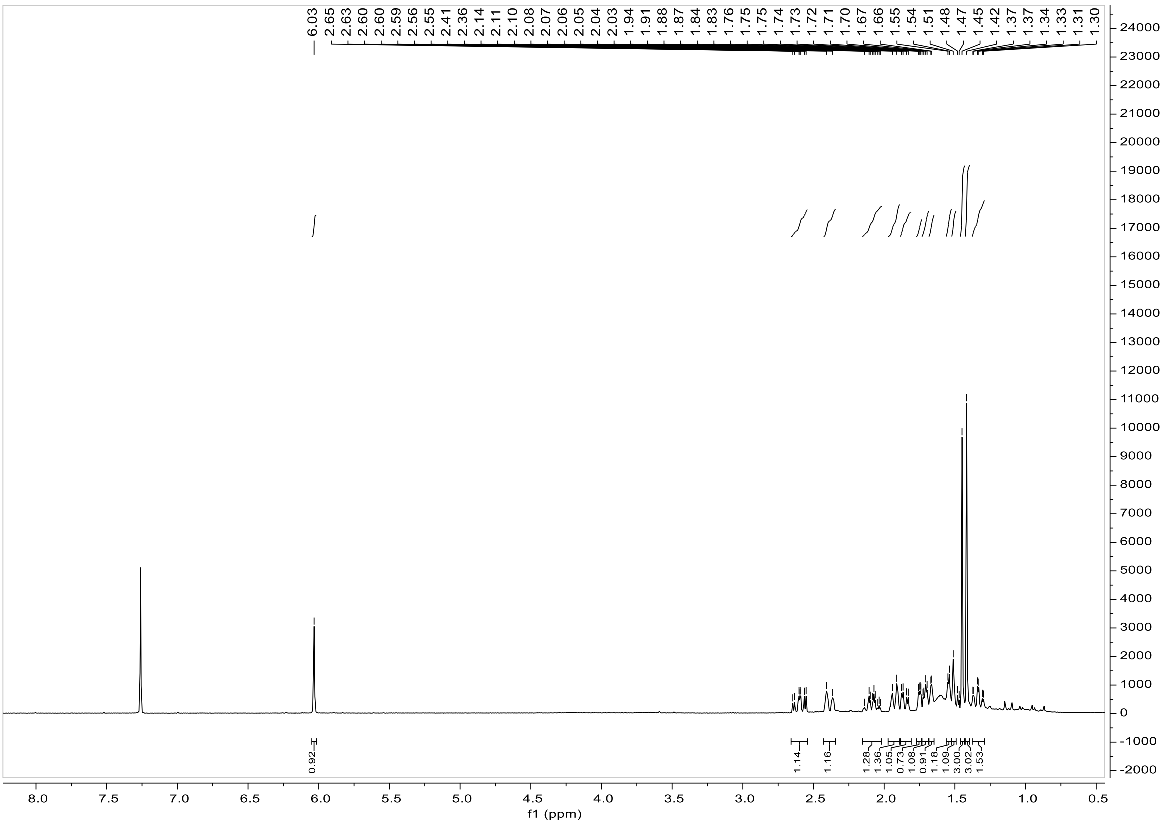

Figure S21. ^{1}H-NMR spectrum of (4*S**,5*E*,10*R**)-7-oxo-tri-nor-eudesm-5-en-4β-ol (**7**) (400 MHz, CDCl$_3$).

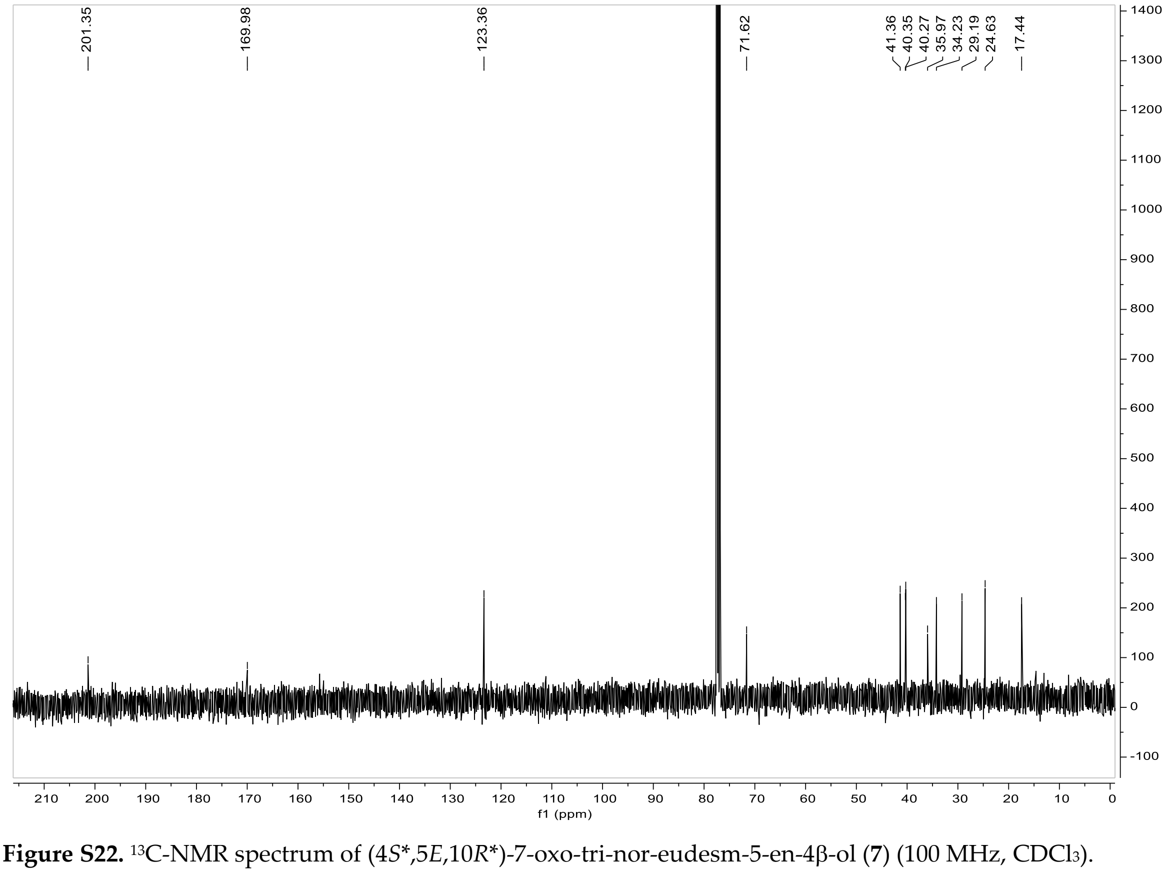

Figure S22. ^{13}C-NMR spectrum of (4*S**,5*E*,10*R**)-7-oxo-tri-nor-eudesm-5-en-4β-ol (**7**) (100 MHz, CDCl$_3$).

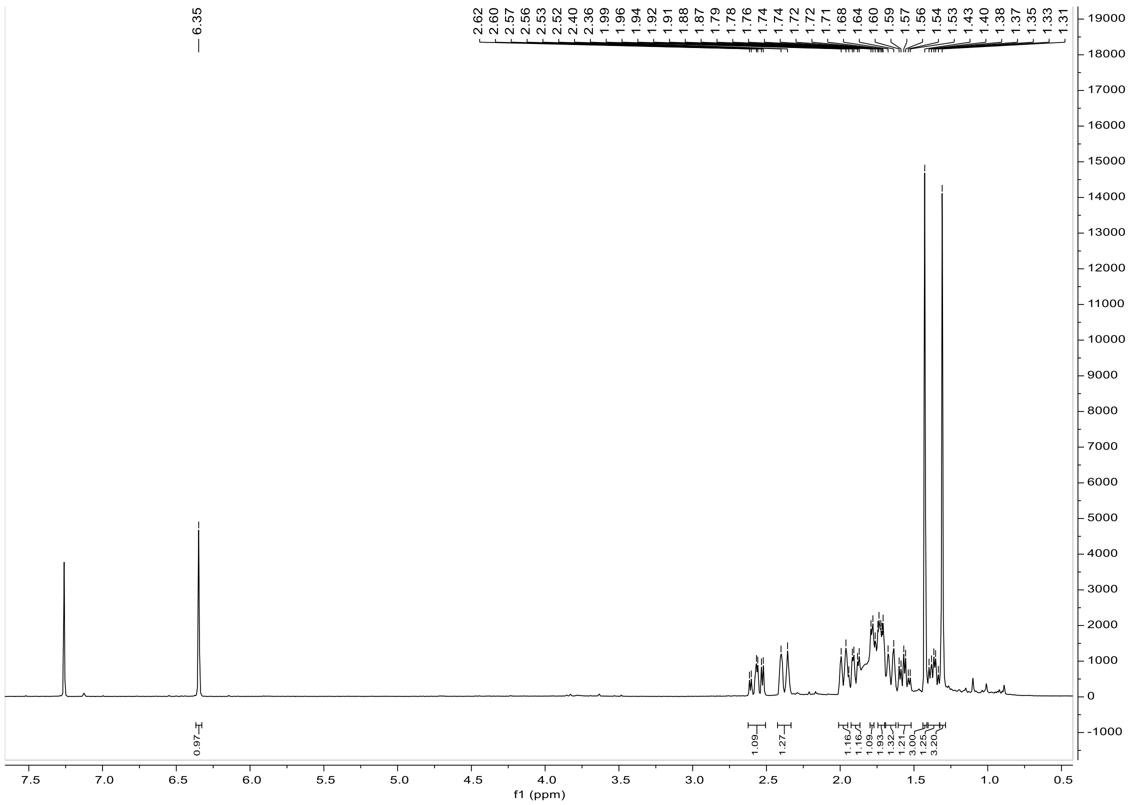

Figure S23. ^{1}H-NMR spectrum of teuhetenone A (**8**) (400 MHz, $CDCl_3$).

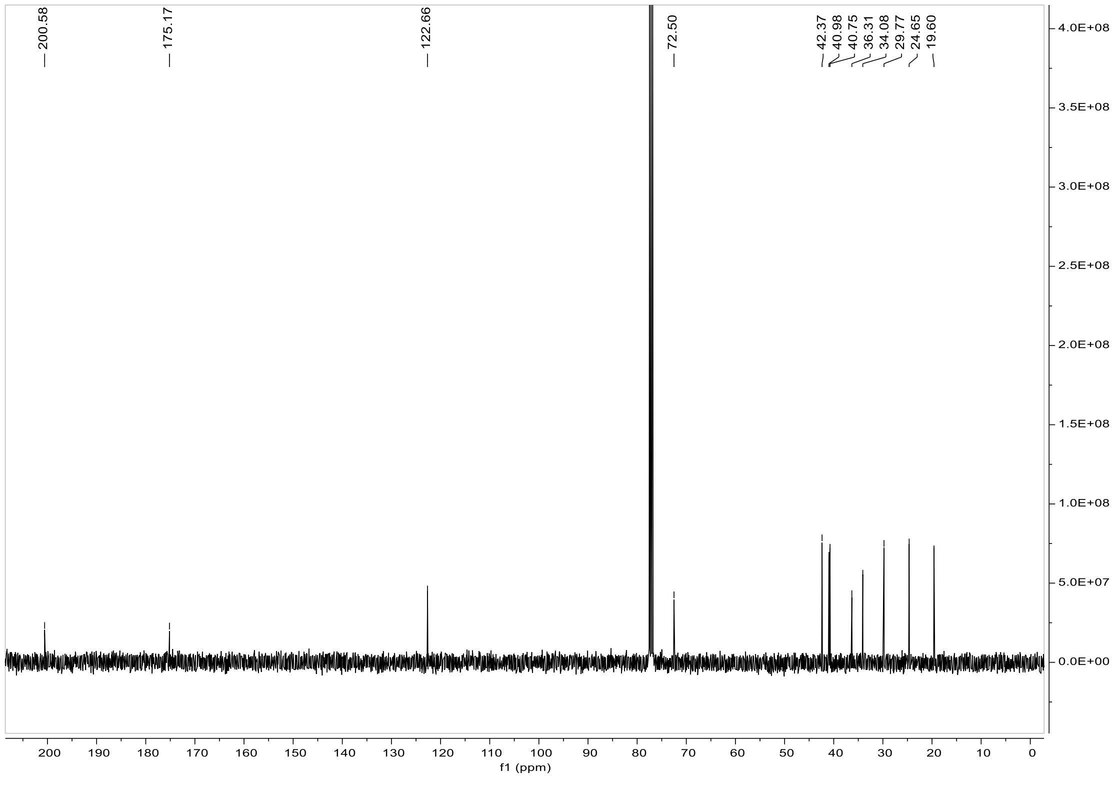

Figure S24. ^{13}C-NMR spectrum of teuhetenone A (**8**) (100 MHz, $CDCl_3$).

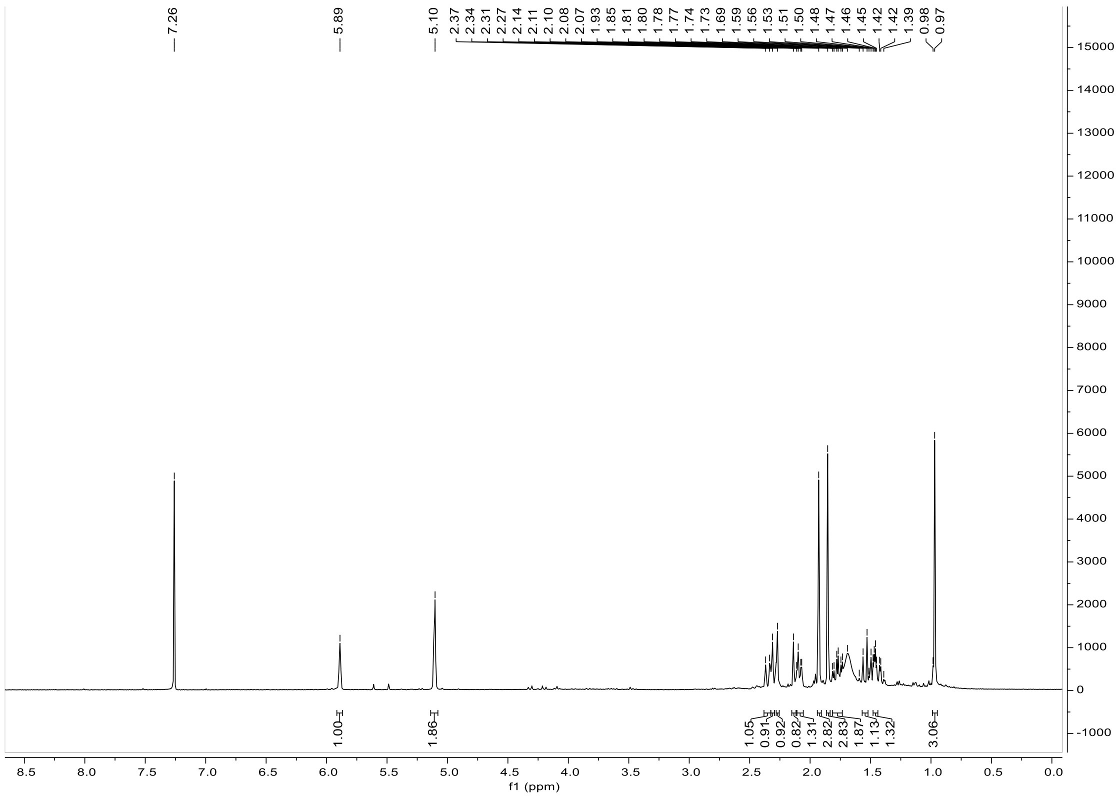

Figure S25. ^{1}H-NMR spectrum of 7-*epi*-teucrenone B (**9**) (400 MHz, $CDCl_3$).

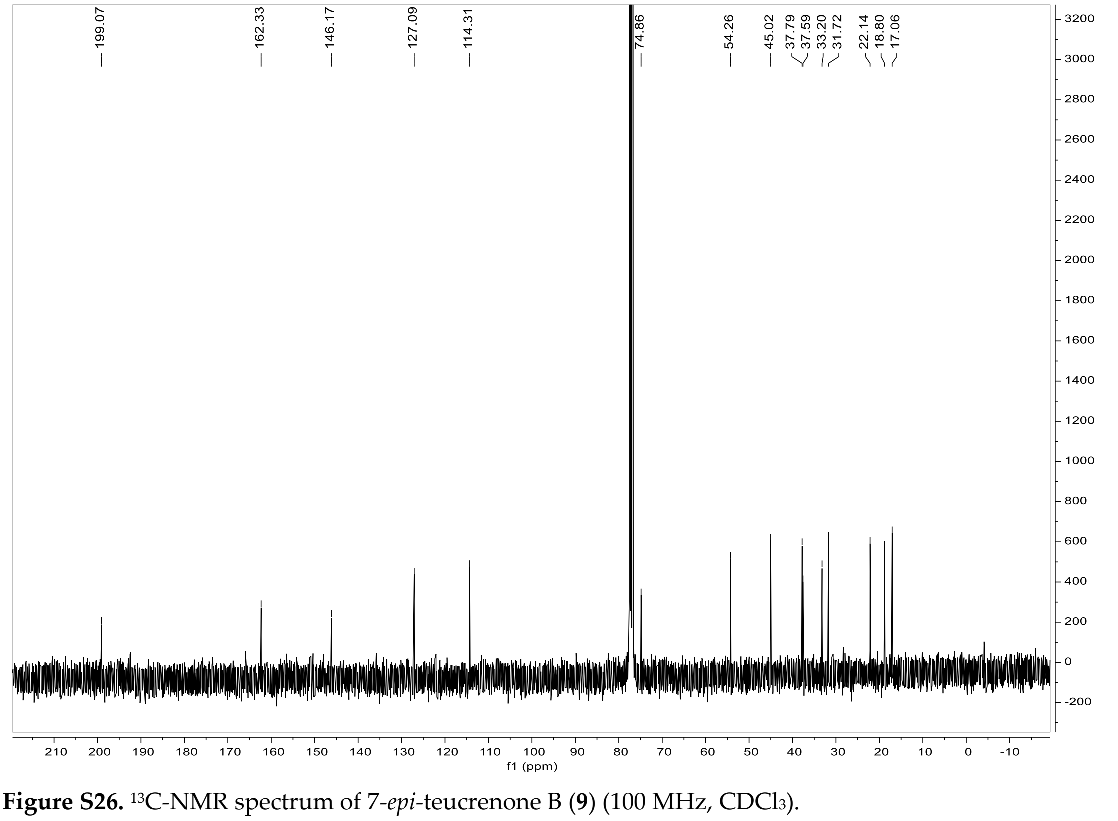

Figure S26. ^{13}C-NMR spectrum of 7-*epi*-teucrenone B (**9**) (100 MHz, $CDCl_3$).

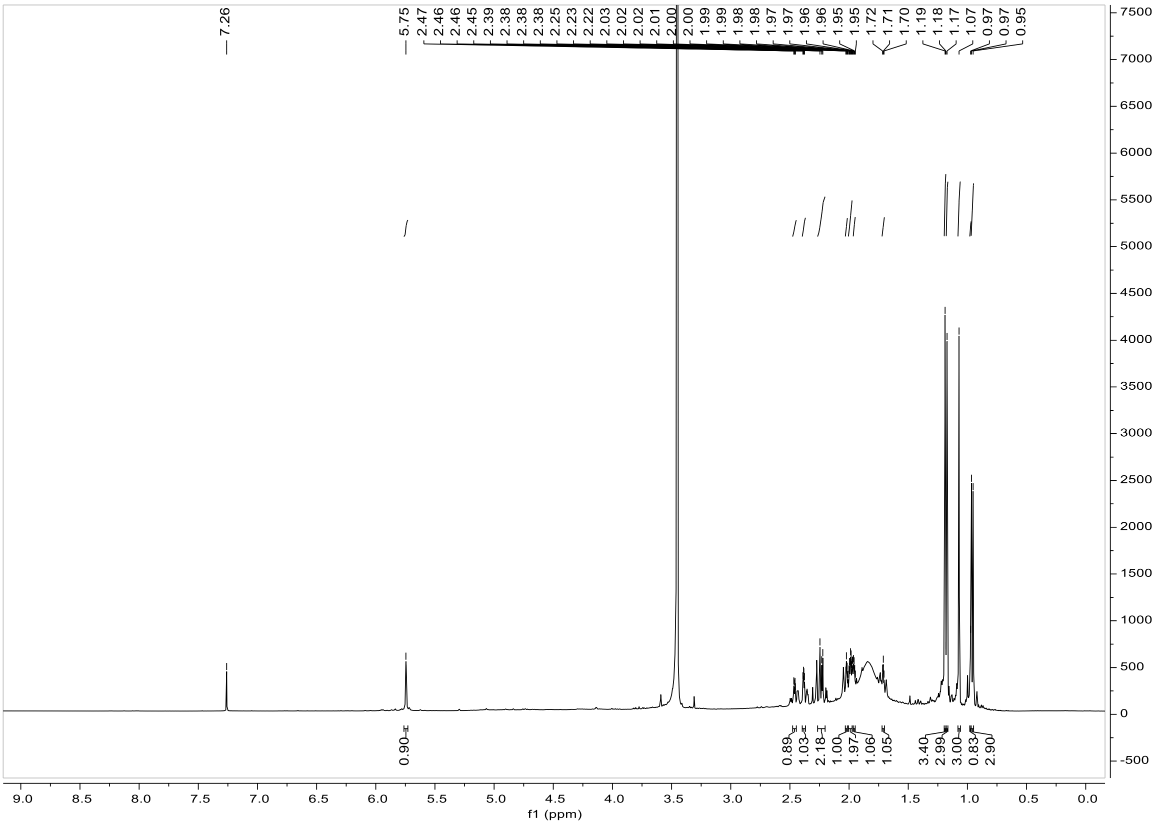

Figure S27. ^{1}H-NMR spectrum of 11-hydroxyvalenc-1(10)-en-2-one (**10**) (400 MHz, $CDCl_3$).

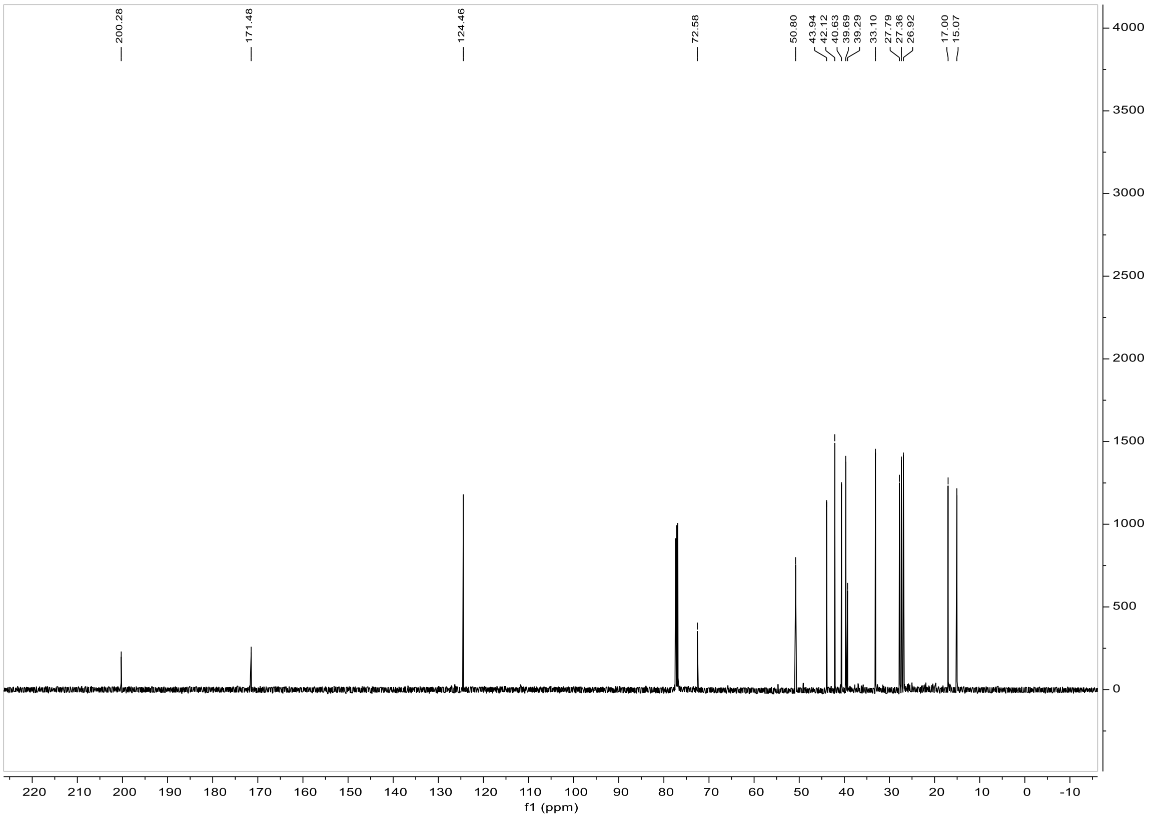

Figure S28. ^{13}C-NMR spectrum of 11-hydroxyvalenc-1(10)-en-2-one (**10**) (100 MHz, $CDCl_3$).

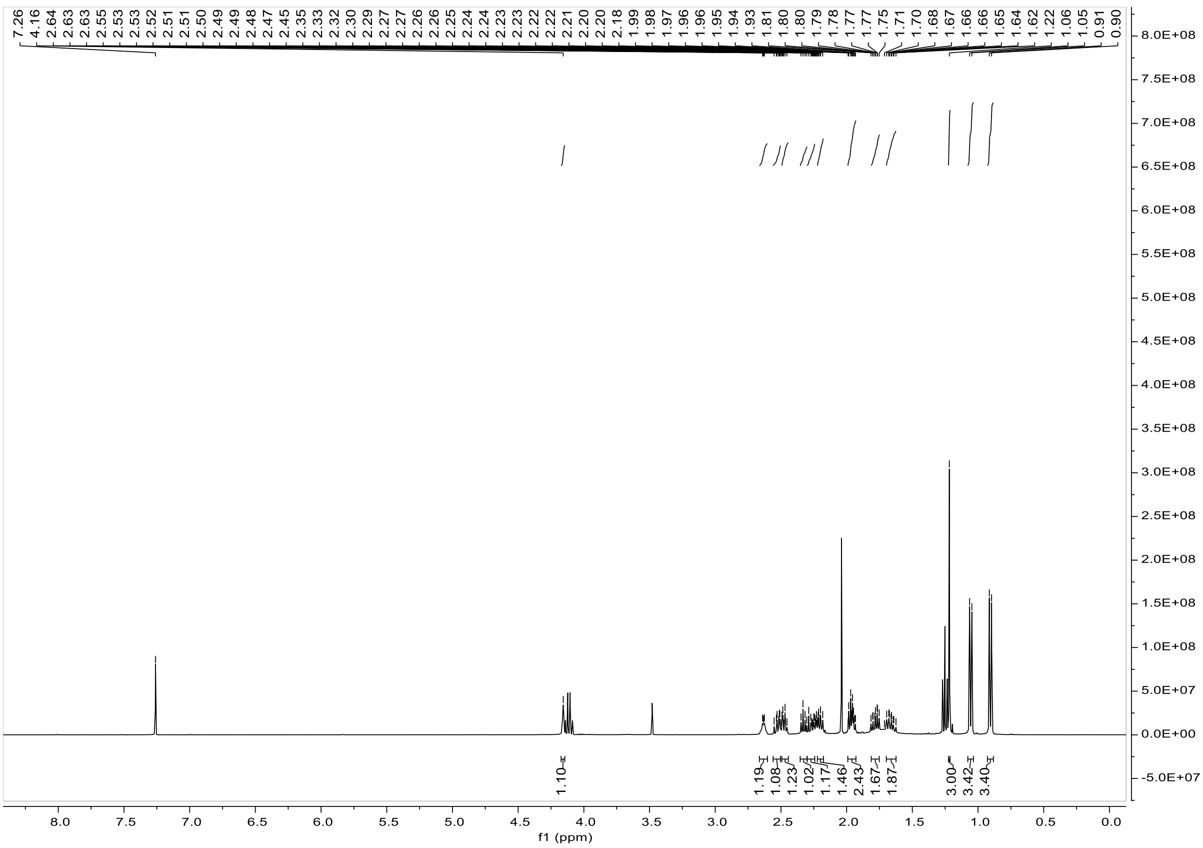

Figure S29. ^{1}H-NMR spectrum of oxyphyllenodiol A (**11**) (400 MHz, $CDCl_3$).

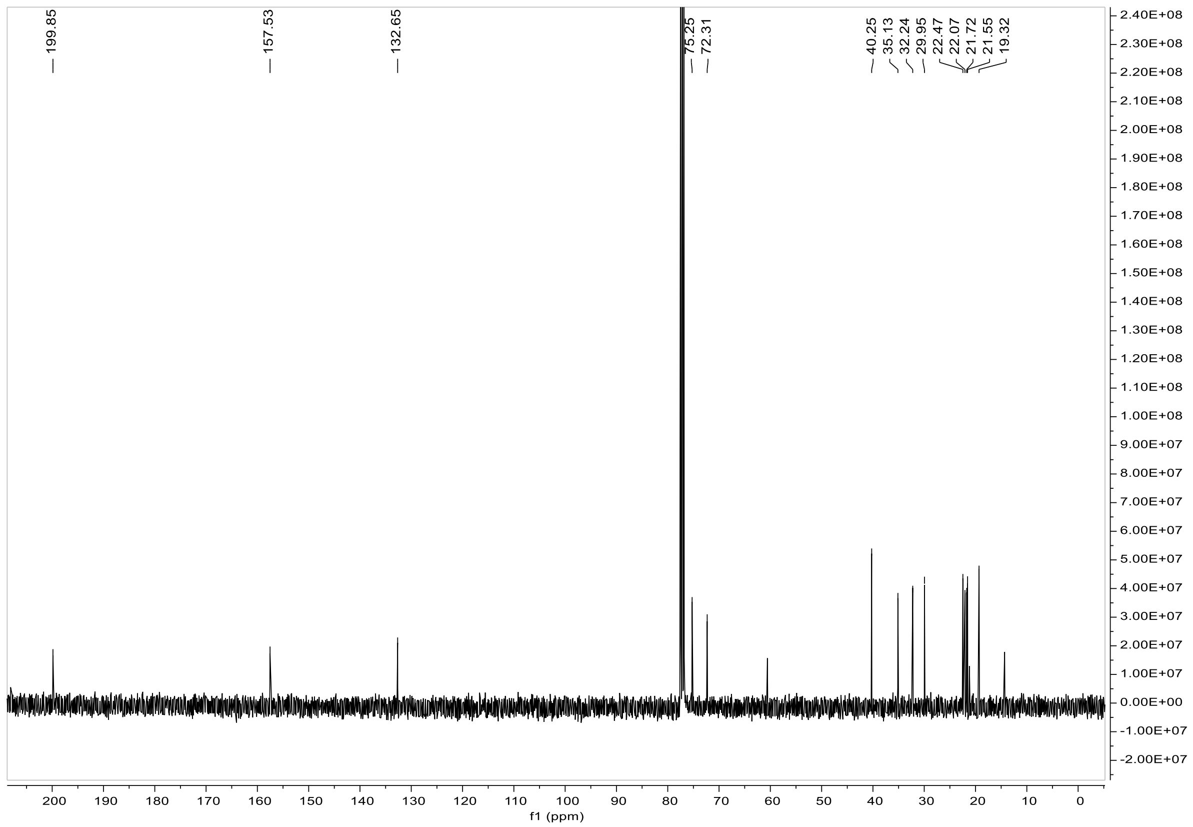

Figure S30. ^{13}C-NMR spectrum of oxyphyllenodiol A (**11**) (100 MHz, $CDCl_3$).

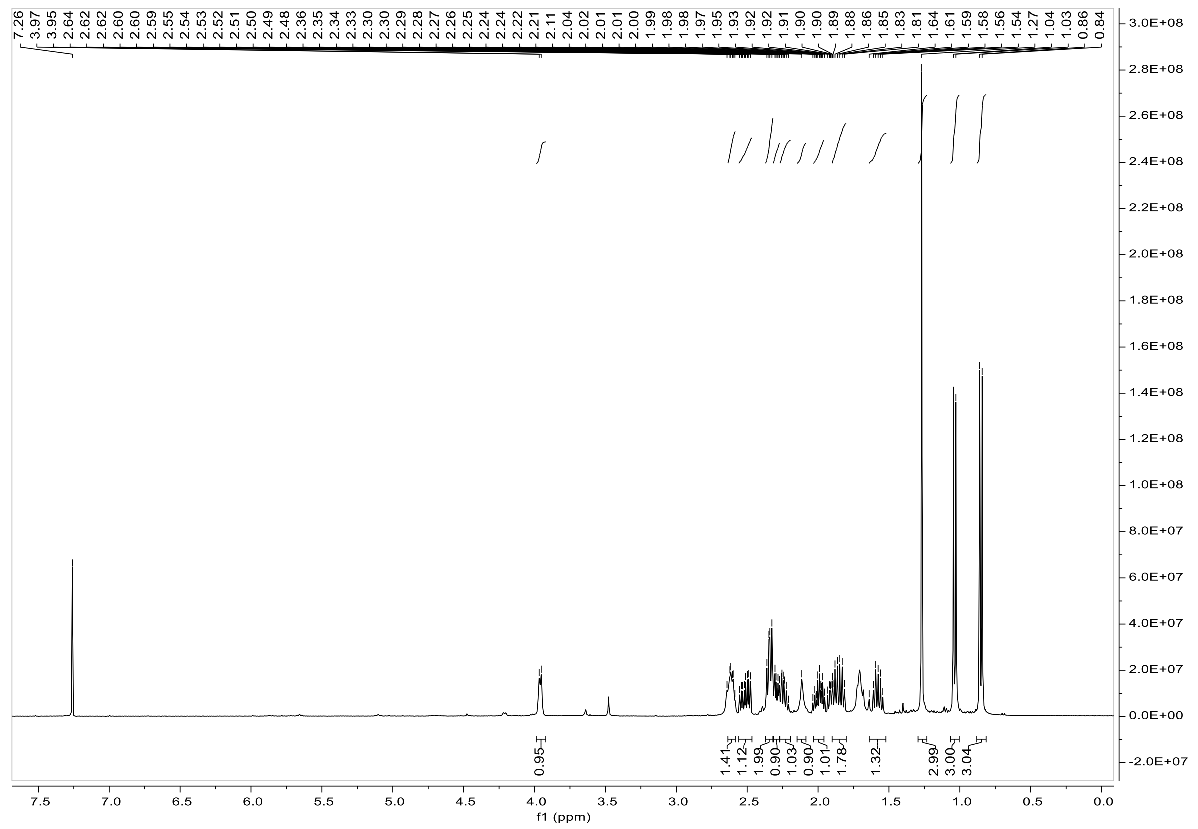

Figure S31. ^{1}H-NMR spectrum of oxyphyllenodiol B (**11**) (400 MHz, $CDCl_3$).

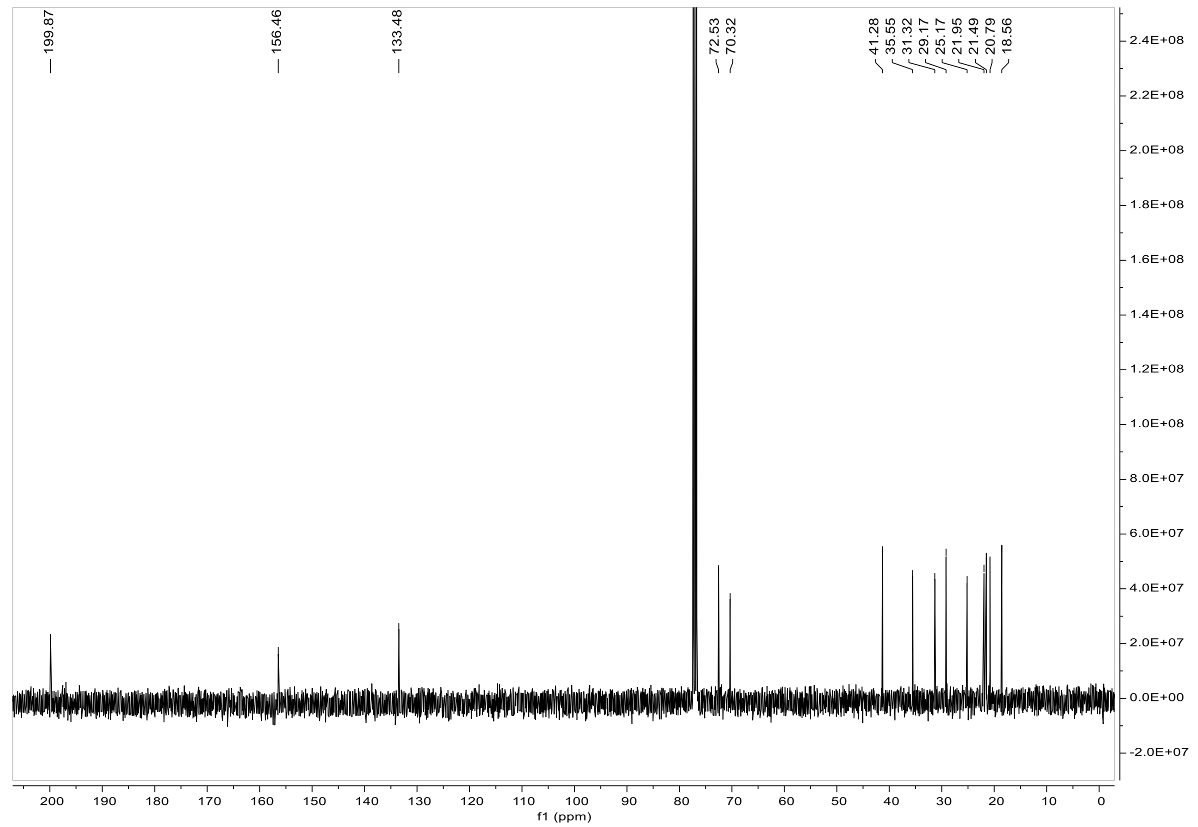

Figure S32. ^{13}C-NMR spectrum of oxyphyllenodiol B (**11**) (100 MHz, $CDCl_3$).

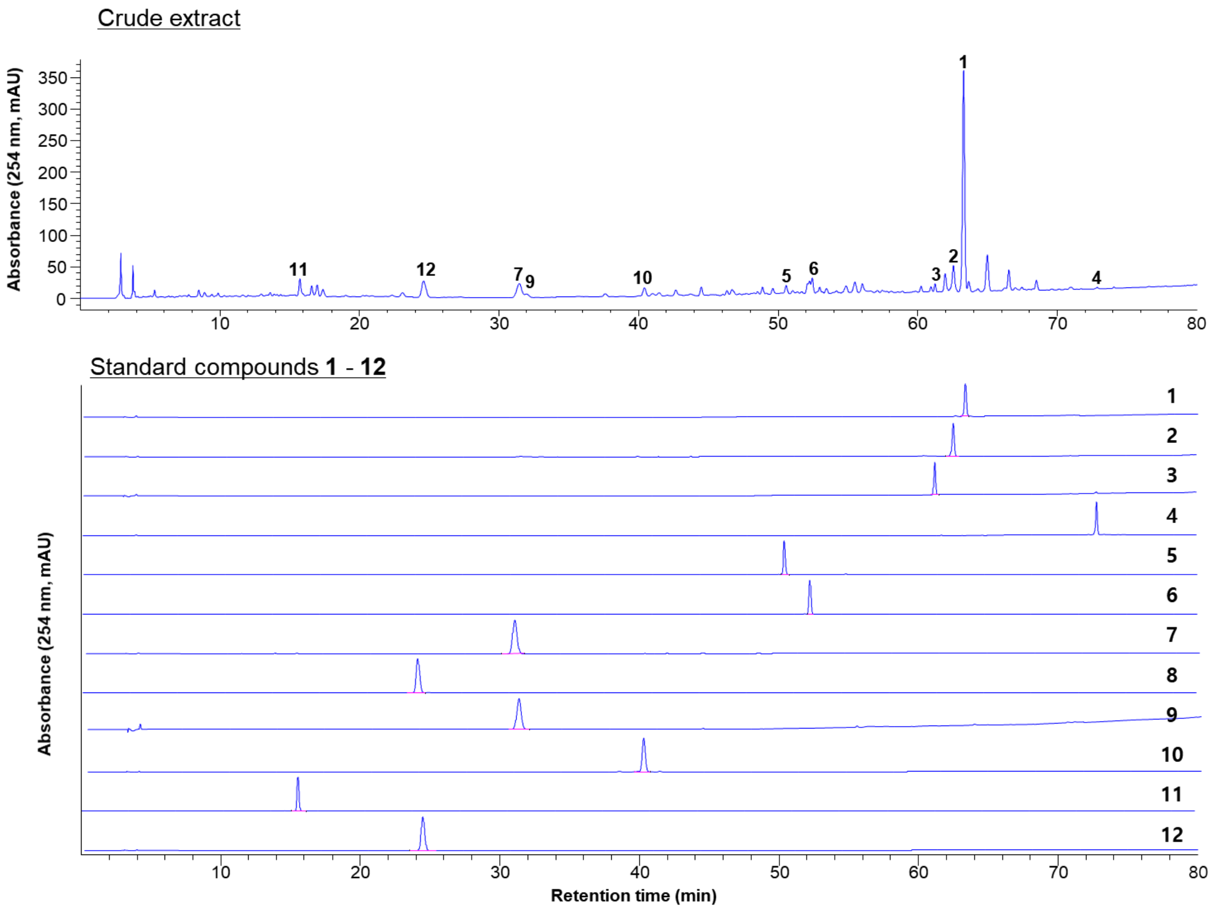

Figure S33. HPLC chromatograms of crude extract and isolated compounds **1** – **12.** Detailed HPLC conditions were described in Materials and Methods section. Isolated compounds: nootkatone (**1**), eudesma-3,11-dien-2-one (**2**), yakuchinone A (**3**), 5'-hydroxyl-yakuchinone A (**4**), alpinenone (**5**), 6α-hydroxy-cyperone (**6**), (4*S*, 5*E*, 10*R*)-7-oxo-tri-nor-eudesm-5-en-4β-ol (**7**), teuhetenone A (**8**), 7-*epi*-teucrenone B (**9**), 11-hydroxyvalenc-1(10)-en-2-one (**10**), oxyphyllenodiol A (**11**), and oxyphyllenodiol B (**12**)

Table S2. Retention time and calibration curves of compounds **1** - **12**.

Compounds	Retention time (min)	Calibration equation	Correlation factor (R^2)
Nootkatone (**1**)	63.3	Y = 18.723X - 341.88	0.9987
Eudesma-3,11-dien-2-one (**2**)	62.5	Y = 14.920X - 66.133	0.9999
Yakuchinone A (**3**)	61.2	Y = 1.2982X + 3.35	0.9998
5'-Hydroxyl-yakuchinone A (**4**)	72.8	Y = 4.297X - 12.025	0.9999
Alpinenone (**5**)	50.5	Y = 10.708X - 130.43	0.9999
6α-Hydroxy-cyperone (**6**)	52.2	Y = 25.533X - 86.608	0.9986
(4*S*, 5*E*, 10*R*)-7-Oxo-tri-nor-eudesm-5-en-4β-ol (**7**)	30.9	Y = 18.057X - 275.68	0.9999
Teuhetenone A (**8**)	24.1	Y = 27.269X - 193.52	0.9997
7-*epi*-Teucrenone B (**9**)	30.9	Y = 0.9248X - 12.3	0.9999
11-Hydroxyvalenc-1(10)-en-2-one (**10**)	40.0	Y = 11.996X - 264.3	0.9991
Oxyphyllenodiol A (**11**)	15.6	Y = 19.116X - 480.89	0.9995
Oxyphyllenodiol B (**12**)	24.5	Y = 12.733X - 59.5	0.9999
